# A positive-feedback loop between HBx and ALKBH5 promotes hepatocellular carcinogenesis

**DOI:** 10.1186/s12885-021-08449-5

**Published:** 2021-06-10

**Authors:** Siming Qu, Li Jin, Hanfei Huang, Jie Lin, Weiwu Gao, Zhong Zeng

**Affiliations:** 1 Organ Transplantation Center, the First Affiliated Hospital of Kunming Medical University, 295 Xichang Road, Kunming, 650032 Yunnan Province China; 2 Institute of Immunology, PLA, Third Military Medical University, 30 Gaotanyan St., District Shapingba, Chongqing, 400038 China

**Keywords:** HBx, ALKBH5, Epitranscriptomics, HCC, m^6^A modification

## Abstract

**Background:**

Hepatitis B Virus (HBV) contributes to liver carcinogenesis via various epigenetic mechanisms. The newly defined epigenetics, epitranscriptomics regulation, has been reported to involve in multiple cancers including Hepatocellular Carcinoma (HCC). Our previous study found that HBx, HBV encodes X protein, mediated H3K4me3 modification in WDR5-dependent manner to involve in HBV infection and contribute to oncogene expression. AlkB Homolog 5 (ALKBH5), one of epitranscriptomics enzymes, has been identified to be associated with various cancers. However, whether and how ALKBH5 is dysregulated in HBV-related HCC remains unclear yet. This study aims to investigate ALKBH5 function, clinical significance and mechanism in HBV related HCC (HBV-HCC) patients derived from Chinese people.

**Methods:**

The expression pattern of ALKBH5 was evaluated by RT-qPCR, Western blot, data mining and immunohistochemistry in total of 373 HBV-HCC tissues and four HCC cell lines. Cell Counting Kit 8 (CCK8) assay, Transwell and nude mouse model were performed to assess ALKBH5 function by both small interference RNAs and lentiviral particles. The regulation mechanism of ALKBH5 was determined in HBx and WDR5 knockdown cells by CHIP-qPCR. The role of ALKBH5 in HBx mRNA N6-methyladenosine (m^6^A) modification was further evaluated by MeRIP-qPCR and Actinomycin D inhibitor experiment in HBV-driven cells and HBx overexpression cells.

**Result:**

ALKBH5 increased in tumor tissues and predicts a poor prognosis of HBV-HCC. Mechanically, the highly expressed ALKBH5 is induced by HBx-mediated H3K4me3 modification of *ALKBH5* gene promoter in a WDR5-dependent manner after HBV infection. The increased ALKBH5 protein catalyzes the m^6^A demethylation of HBx mRNA, thus stabilizing and favoring a higher HBx expression level. Furthermore, there are positive correlations between HBx and ALKBH5 in HBV-HCC tissues, and depletion of ALKBH5 significantly inhibits HBV-driven tumor cells’ growth and migration in vitro and in vivo*.*

**Conclusions:**

HBx-ALKBH5 may form a positive-feedback loop to involve in the HBV-induced liver carcinogenesis, and targeting the loop at ALKBH5 may provide a potential way for HBV-HCC treatment.

**Supplementary Information:**

The online version contains supplementary material available at 10.1186/s12885-021-08449-5.

## Background

Cancer is the epigenetic disease with hallmarks of irregular DNA methylation, disrupted chromatin state and dysregulated non-coding RNA [1, 2]. HBV infection is the primary reason in East-Asian [3] and it promotes tumorigenesis by inducing various epigenetics hallmarks [4, 5]. The common mechanism is that HBV dysregulates or hijacks host epigenetic enzymes such as DNA methylase, histone methylase or acetylase, to induce aberrant epigenetic instability of host cells [6, 7]. Our previous study found that the X protein of HBV (HBx) mediated a higher level of epigenetic H3K4me3 modification of both host and virus chromatin via up-regulation of WD-40 Repeat Protein 5 (WDR5), the core subunit of histone modification enzyme, thus promoting HBV infection and contributing to hepatocellular carcinogenesis [6]. However, it’s still unclear and incomprehensive how the epigenetic dysregulation occurs during HBV infection, which hinders the completely overcome of HBV-induced pathology of liver.

A new layer of epigenetic regulation, epitranscriptomics, has been added by recent studies [8], which means chemical modifications on cellular RNA instead of on DNA or histone compared to the ‘epigenetics’ term. Epitranscriptomics contain more than 140 RNA modifications, and m^6^A modification is the most prevalent of eukaryotic cells and plays critical roles in many biological and pathological contexts [9]. The m^6^A modification is mainly deposited at the RR(m^6^A) CH motif of mRNA and recognized by its catalytic enzymes, thus regulating mRNA stability, transportation, translation and turnover [10, 11]. Our previous study found that both HBx and WDR5 bind to the promoter of AlkB Homolog 5 (ALKBH5) with the H3K4me3 modification [6]. ALKBH5, the demethylase enzyme, has been reported to be highly expressed in various cancers including Hepatocellular Carcinoma (HCC), suggesting its common role in tumor genesis and progression [12, 13]. In addition, HBV has been reported to hijacks the epitranscriptomics enzymes to induce either increased or decreased m^6^A modification of cognate mRNA including itself encoding mRNA [14, 15]. However, whether and how ALKBH5 is involved in HBV related HCC (HBV-HCC) patients derived from Chinese people remains to be elucidated yet.

In the present study, we demonstrated that ALKBH5 is highly expressed and correlated to a poor prognosis in HBV-HCC patients. Furthermore, we observed HBV up-regulates ALKBH5 via the HBx-WDR5-H3K4me3 axis, and ALKBH5 forms a positive feedback loop with HBx to lead to hepatocellular carcinoma progression. The results suggest HBV may hijacks the epitranscriptomics enzyme ALKBH5 to induce HCC through the HBx-ALKBH5 positive feedback, which might represent a novel therapeutic target of HBV-HCC.

## Methods

### Patients and HCC specimens

This study was approved by the Ethical Committee of the First Affiliated Hospital of Kunming Medical University (Clinical Ethic Issue No: kmmu2019483) following guidelines of the Declaration of Helsinki. All the participants signed written informed consents, and they gave consent to have their data published. Twenty pairs of fresh HBV-HCC tissues with matched peri-tumor tissues from Chinese patients who were diagnosed as HBV-related primary HCC and normal liver tissues from patients with repair treatment of traumatic hepatic rupture were collected from the First Affiliated Hospital of Kunming Medical University to test the ALKBH5 mRNA and protein expression level. Seventy-nine pairs of Chinese HBV-HCC tissues and paried peritumor tissues from commercial tissue microarrays supplied by Shanghai Superbiotech (LD-LVC1805, Shanghai, China) were processed into immunohistochemical (IHC) staining together with integrated follow-up and clinical information data.

### Cell culture and animal studies

The human HCC cell lines L02, HepG2, MHCC97H, HepG2.2.15 were from ATCC (Manassas, USA). The primary human hepatocytes (PHH) were purchased from the iCELL company (Shanghai, China) and the HepG2-NTCP cells were previously constructed by our institute. The stable ALKBH5-knockdown (KD) cell lines of HepG2.2.15 were generated by lentivirus infection with puromycin selection according to the manufacturer’s protocol (Santa Cruz). Male athymic BALB/c nude mice (4 weeks old) were used for tumor formation assay. The animal experiments were approved according to the guidelines of the Animal Care and Use Committee of the First Affiliated Hospital of Kunming Medical University (IACUC Issue No: kmmu2019370), and the study was carried out in compliance with the ARRIVE guidelines. HepG2.2.15 cells with ALKBH5-KD or control were prepared in PBS and injected subcutaneously with 1 × 10^7^ cells of the nude mice. After 3 weeks, tumors were excised to measure weight.

### HBV infection and plasmid transfection

HBV infection assay was performed according to previous reports [16]. Briefly, plasmid of wildtype HBV 1.1mer (carrying a greater-than-unit-length HBV genome) or mutation of HBV 1.1mer with HBx abrogation (the same plasmid with a stop codon for amino acid 7 of HBx) were transiently transfected into HepG2 cells and the supernatants were collected by polyethylene glycol (PEG) 8000 and centrifugated to enrich the viral particles. For the following HBV infection of HepG2-NTCP or PHH cells, media were supplemented with final 4% PEG-8000 plus HBV particles and added to the cells with 50–80% confluency, 3 days after HBV infection, cells were collected to conduct related experiments. Silencing RNA oligos for knockdown of HBx or WDR5 were synthesized according to our published literature [6]. The wild-type HBx 3’UTR and the mutated HBx 3’UTR (A1907C mutation, diminishing the site of m^6^A modification), namely HBx 3’UTR WT and HBx 3’UTR MT, respectively, overexpressing plasmids were constructed based on endonuclease digestion and T4 DNA ligation. The primers used in these procedures are listed in Table 1. RNA oligos or plasmids were transfected into cells using Lipofectamine 3000 reagent (Thermo Fisher Scientific, USA) and cells were collected after 48 h to be used in the related experiments.

**Table 1:** Primers used in this study

Application and amp icon	Sequence (5′ → 3′)
**Oligos for RT-qPCR**	
GAPDH-qpcr-F	AACGGATTTGGTCGTATTGGGC
GAPDH-qpcr-R	TTCGCTCCTGGAAGATGGTGAT
HBx-qpcr-F	CCGTCTGTGCCTTCTCATCTGC
HBx-qpcr-R	ACCAATTTATGCCTACAGCCTCC
ALKBH5-qpcr-F	GGCCTCAGGACATCAAGGAGC
ALKBH5-qpcr-R	ACAGGGACCCTGCTCTGAAAC
**siRNA for knockdown**	
siWDR5^a1^	CACCUGUGAAGCCAAACUU
siHBx^b2^ (duplex mixture)	GCAUACUUCAAAGACUGUUUG GGUUAAAGGUCUUUGUACUAG GGCAUAAAUUGGUCUGUUCAC GUUUUUCCCCUCUGCCUAA
**Oligos for RT-qPCR**	
ALKBH5-TSS-F1	AAATATTCGGACGATGCCGTGACGCGG
ALKBH5-TSS-R1	TTTATACGGGCATGCGCGTGCGTGCA
ALKBH5-TSS-F2	TATAGGACCCTAGAGCAGCGTCGT
ALKBH5-TSS-R2	AAATATGTGTCCGGGGCCAAGCG
**Oligos for MeRIP-qPCR**	
HBx-3’UTR-F	CCGTCATCTCTTGTTCATGTCC
HBx-3’UTR -R	CGGCCGCTCCAAATTCTTTATAAGGG
**Oligos for plasmids construction**	
EcoRI-HBx-F	GGTGAATTCATGGCTGCTAGGCTGTGCTGC
NotI-HBx 3UTR WT-R	CTTAGCGGCCGCTCCAAATTCTTTATAAGGGTCG
NotI-HBx 3UTR A1907C-R2	TTAGCGGCCGCTCCAAATTCTTTATAAGGGTCGATGGCCATGCCCCAAAGCCACCC

^a^1, the siWDR5 RNA oligo are synthesized according to this paper: *X-Linked Mental Retardation Gene Product CUL4B Targets Ubiquitylation of H3K4 Methyltransferase Core Component WDR5 and Regulates Neuronal Gene Expression.* Mol Cell. 2011 Aug 5; 43 (3): 381–391

^b^2, the siHBx RNA oligo are synthesized according to this paper: *Therapeutic recovery of hepatitis B virus (HBV)-induced hepatocyte-intrinsic immune defect reverses systemic adaptive immune tolerance.* Hepatology. 2013 Jul;58 (1):73–85

### RT-qPCR, Western blot and IHC

RT-qPCR, Western blot and IHC were performed according to the previous reports [6, 17]. Antibodies: anti-ALKBH5 and anti-HBx antibodies (Abcam, USA) and anti-β-actin antibody (Cell Signaling Technology, USA). Primers sequences used for RT-qPCR were included in Table 1.

### ChIP-qPCR

The ChIP assay was performed as previously described [18]. In brief, final 1% concentration formaldehyde fixed chromatin from 10 million HepG2.2.15 cells with different treatment and were sonicated to about 500 bp fragments, antibody-bound protein A/G Dynabeads (Invitrogen) were incubated with sonicated chromatin at 4 °C overnight. After 4 rounds of washing, the precipitated protein-DNA complex was reverse-crosslinked at 65 °C overnight. DNA were purified by Phenol chloroform. The ChIP-qPCR primers used in this study are presented in Table 1. Antibodies used in ChIP were H3K4me3 (ab8580, Abcam).

### Cell proliferation and Transwell assay

Cell proliferation was assessed by cell counting kit 8 (CCK8) assay (Beyotime, China). In brief, cells were seeded onto 96-well plates. CCK8 solution (10 μL/well) was added to the cells after incubating for the indicated time points, and the reaction product was quantified according to the manufacturer’s instructions. Transwell assays were performed using 24-well Transwell plates (8-μm pore size; Millipore, Burlington, MA, USA). 1 × 10^5^ cells were seeded in serum-free medium in the upper chamber, whereas medium supplemented with 20% fetal bovine serum was applied to the lower chamber as a chemoattractant. After 48 h of incubation, the migrated cells at the bottom surface of the filter were fixed, stained, and counted.

### MeRIP-seq and MeRIP -qPCR

The MeRIP assay was conducted following the manufacturer’s procedure (Merck Millipore). In brief, total RNA were purified by TRIzol reagent (Japan, TAKARA) and mRNA were enriched and fragmented by the commercial kit (Invitrogen). m^6^A antibody conjugated proteinA/G Dynabeads were incubated with mRNA at 4 °C for 4 h. After washing, the precipitated mRNA was submitted to library construction or RT-qPCR assay. MeRIP-qPCR primers used in this study are presented in Table 1.

### Bioinformatics analysis

RNA-seq and Mass spectrum (MS) data of 159 liver samples (paired tumor and peri-tumor) from Fudan Chinese HBV-HCC data was downloaded through the URL: https://www.biosino.org/node/project/detail/ OEP000321. RNA-seq data of 115 HBV-HCC samples from The Cancer Genome Atlas (TCGA) were downloaded from the URL: https://cbioportal-datahub.s3.amazonaws.com/lihc_tcga.tar.gz and the HBV infection and genotype information was based on *ViralMine* analysis [19]. MeRIP-seq and input RNA were sequenced and mapped to human genome using HISAT2 (http://daehwankimlab.github.io/hisat2). The m^6^A peaks were called by exomePeak (https://bioconductor.org/packages/exomePeak).

### Statistical analysis

All statistical analysis was performed using GraphPad Prism 8 software (GraphPad Software, La Jolla, CA, USA). Unpaired or paired two-tailed Student’s *t*-test were applied to assess comparison between two groups; Welch’s ANOVA test was used for comparison among groups; Kaplan-Meier survival curves and log-rank tests were used to estimate survival and differences between groups; The Pearson’s chi-square test was used for correlation analysis with calculation of correlation coefficients (r); The statistical significance was indicated by *, *P* < 0.05; **, *P* < 0.01; ***, *P* < 0.001; ****, *P* < 0.0001 and not significant (NS) when *P* > 0.05.

## Result

### ALKBH5 is highly expressed in HBV-HCC and predicts poor prognosis

**Fig. 1:**
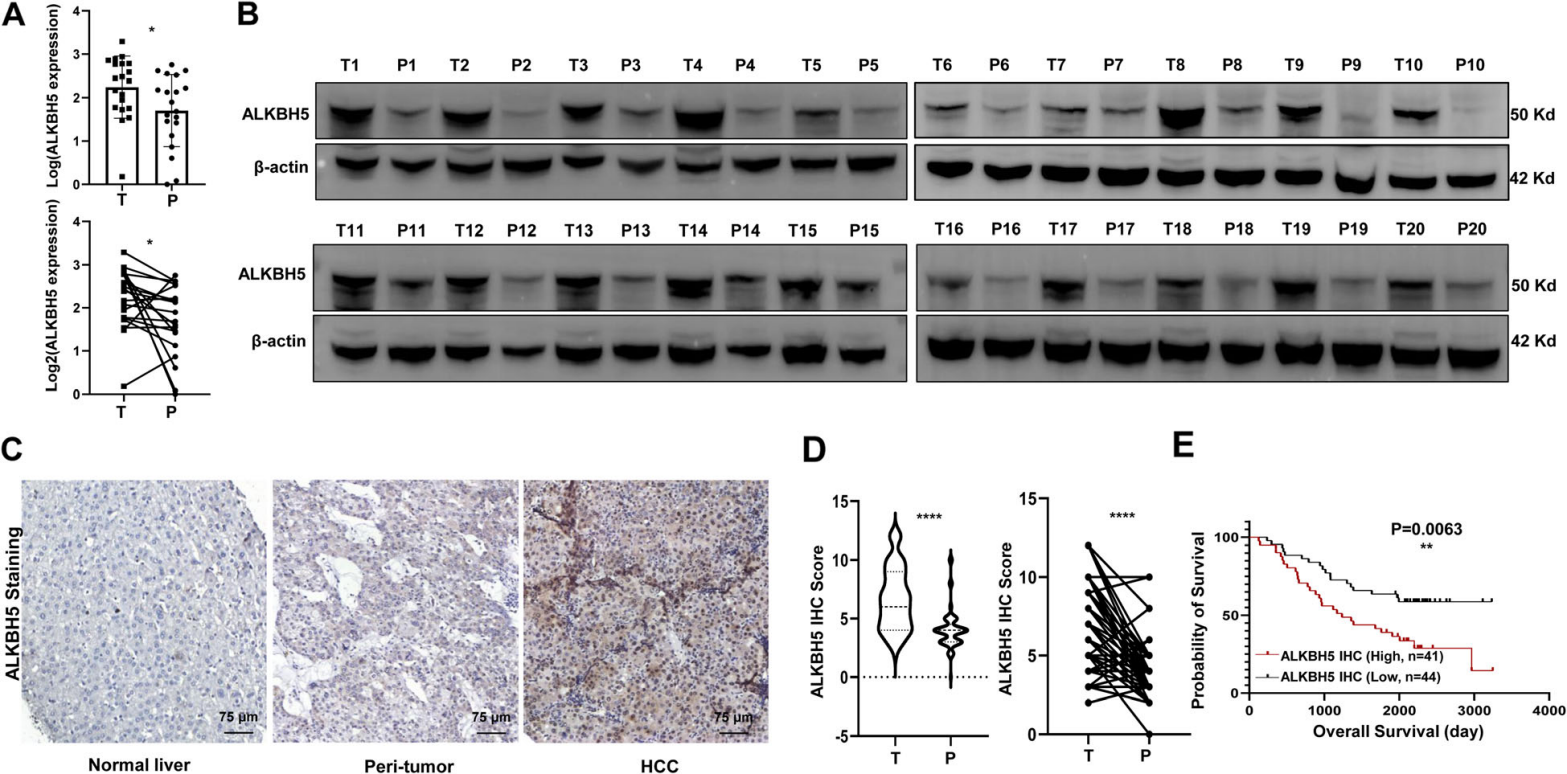
The mRNA and proterin level of ALKBH5 are highly expressed in HBV-HCC tissue. **A**The mRNA and **B** protein levels of ALKBH5 in the indicated HBV-HCC or matched peri-tumor tissues were determined by RT-qPCR (*n* = 20) and Western blot assays (*n* = 20), respectively. GAPDH was used as the internal control in RT-qPCR assay and β-actin was used as the normalized control in Western blotting assay. **C** Representative images of ALKBH5 IHC staining in normal liver, peri-tumor and tumor tissue of HBV-HCC patients (magnification 200×). **D** ALKBH5 IHC score in tumor and paired peri-tumor from 79 HBV-HCC tissue microarray. **E** Kaplan-Meier analysis of ALKBH5 IHC score and overall survival from 79 HBV-HCC tissue microarray. T, tumor; P, peri-tumor

Several studies reported the increased ALKBH5 in HCC based on TCGA dataset [20, 21] while other reports indicated decreased ALKBH5 in HCC [22, 23], suggesting ALKBH5 might function differently in different HCC background (such as HBV versus non-HBV related HCC). To define ALKBH5 expression pattern in HBV-HCC, we collected 20 pairs of tumor and peri-tumor tissues with HBV infection history from Chinese patients. ALKBH5 mRNA and protein levels were significantly increased in HCC tissues compared to peri-tumor (Fig. 1A and B). IHC assay further confirmed that ALKBH5 was mainly stained in the nuclei of tumor cells and there was the highest expression of ALKBH5 in tumor cells compared with moderate expression in the peritumor tissues and the negative staining in normal liver tissues (Fig. 1C). We further use a tissue microarray including another 79 pairs of tumor and peri-tumor from Chinese HBV-HCC patients to assert the ALKBH5 expression pattern. The IHC score of ALKBH5 was predominantly higher in tumors than their paired peri-tumors from the 79 Chinese HBV-HCC patients (Fig. 1D). And the higher IHC score of ALKBH5 in HBV-HCC tissue microarray predicted a poor prognosis (Fig. 1E).

**Fig. 2:**
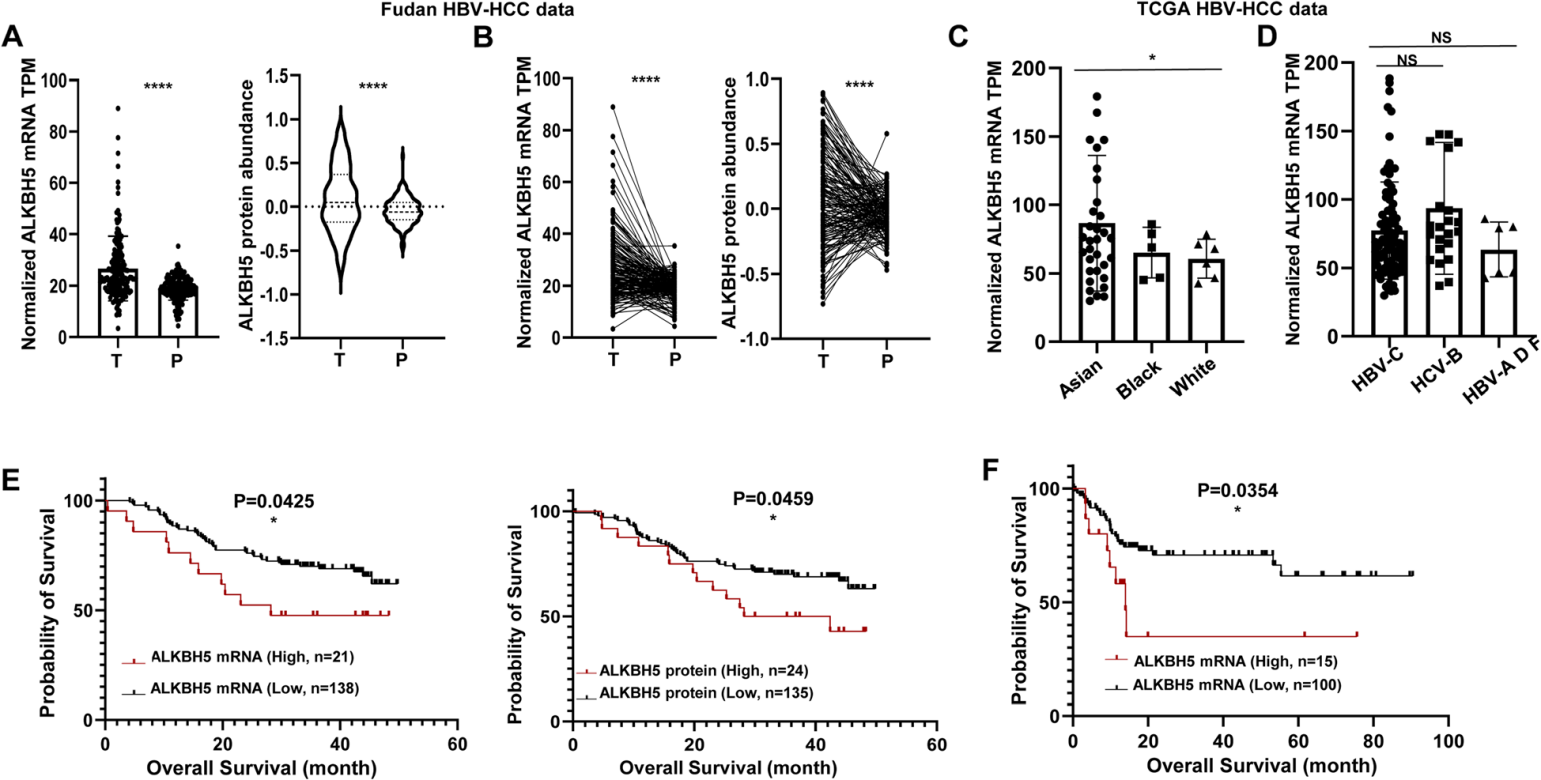
ALKBH5 is predominately increased in 274 HBV-HCC patients and predicts poor prognosis. **A**, **B** ALKBH5 mRNA TPM score and protein abundance in tumor and paired peri-tumor from 159 Fudan HBV-HCC patients’ transcriptome and proteome dataset. **C** ALKBH5 mRNA TPM score in tumors of Asian, Black and White ethnicity from 115 TCGA HBV-HCC patients’ transcriptome. **D** ALKBH5 mRNA TPM score in tumors of HBV C, B and other genotypes from 115 TCGA HBV-HCC patients’ transcriptome. **E** Kaplan-Meier analysis of ALKBH5 mRNA TPM, protein abundance and overall survival of 159 Fudan HBV-HCC patients. **F** Kaplan-Meier analysis of ALKBH5 mRNA TPM and overall survival of 115 TCGA HBV-HCC patients. T, tumor; P, peri-tumor

To expand the sample size and dissect ALKBH5 role in HBV-HCC patients, we data mined a recently comprehensive Fudan university’s study of HBV-HCC including 159 Chinese patients’ transcriptome and proteome [24]. The ALKBH5 mRNA and protein level in tumors and peri-tumors from each of 159 Chinese patients were extracted to assess ALKBH5 expression pattern. The average mRNA and protein level of ALKBH5 were significantly higher in tumors than in peri-tumors of the 159 Chinese patients (Fig. 2A). 115/159 of Fudan HBV-HCC samples displayed a higher ALKBH5 mRNA level, 96/159 for higher ALKBH5 protein level, in tumor compared to paired peri-tumor tissues and there were significant differences by paired *t* test (Fig. 2B). Considering that Fudan HBV-HCC samples come all from Chinese patients and HBV genotype B and C are more popular in China than other genotypes [25], we further data mined TCGA HBV-HCC datasets to explore whether the expression level of ALKBH5 in the HBV background is related to different ethnicity or different HBV genotypes. We found that the ALKBH5 level was higher in Asian groups than White and Black groups (Fig. 2C); However, there’s no significant difference of the ALKBH5 expression level among HBV genotypes B, C, and other phenotypes (Fig. 2D). These TCGA HBV-HCC results suggest that the ALKBH5 expression might be correlated to ethnicity but not the HBV phenotype.

Survival analysis of both Fudan and TCGA HBV-HCC datasets indicated that patients with higher mRNA and protein levels of ALKBH5 had markedly less total survival rate than patients with lower ALKBH5 expression (Fig. 2E and F). Furthermore, significant positive associations of ALKBH5 expression were identified with gender, tumor size, AFP level, TNM stage and HCC proliferation subtype, whereas there was no significant association with age, liver cirrhosis, tumor encapsulation and tumor number (Table 2). Taken together, ALKBH5 was highly expressed in most HBV-HCC tissues and could predict poor prognosis for HBV-HCC patients.

**Table 2:** Relationship between clinicopathologic characteristics and ALKBH5 mRNA and protein level in 159 cases of HBV-HCC cohort

Characteristics	ALKBH5 mRNA			ALKBH5 protein		
	High	Low	*P* value	High	Low	*P* value
**Gender**						
male	59	69	0.0657	58	70	**0.025**
female	20	11		21	10	
**Age**						
> = 54	38	46	0.2352	39	45	0.3847
< 54	41	34		40	35	
**Cirrhosis**						
yes	55	57	0.8218	56	56	0.9026
no	24	23		23	24	
**Tumor number**						
> 1	22	20	0.6838	21	21	0.9621
1	57	60		58	59	
**Tumor size (cm)**						
> = 5.5CM	48	35	**0.0318**	46	37	0.1306
< 5.5CM	31	45		33	43	
**Tumor thrombus**						
yes	22	15	0.1746	19	18	0.817
no	57	65		60	62	
**Tumour enapsulation**						
yes	54	57	0.6909	57	54	0.5229
no	25	23		22	26	
**AFP**						
> 1000	29	21	0.1556	33	17	**0.0053**
< 1000	50	59		46	63	
**BCLC stage**						
A	30	38	0.2134	30	38	0.4787
B	25	27		28	24	
C	24	15		21	18	
**TNM stage**						
I + II	46	59	**0.0388**	50	55	0.4674
III + IV	33	21		29	25	
**Proteomic subtype**						
1(S-Mb)	22	33	0.1832	17	38	**0.0027**
2(S-Me)	30	27		34	23	
3(S-Pf)	27	20		28	19	
**mRNA subtype**						
1(S-Mb)	17	42	**0.0003**	20	39	**0.0084**
2(S-Me)	33	21		33	21	
3(S-Pf)	29	17		26	20	

ALKBH5 expression from the dataset (Cell. 2019 Nov 14;179 (5):1240) was divided into low and high expression groups based on the medium of mRNA TPM score or protein abundance score, respectively. AFP, Alpha Fetoprotein

### HBV up-regulates ALKBH5 via the HBx-WDR5-H3K4me3 axis

**Fig. 3:**
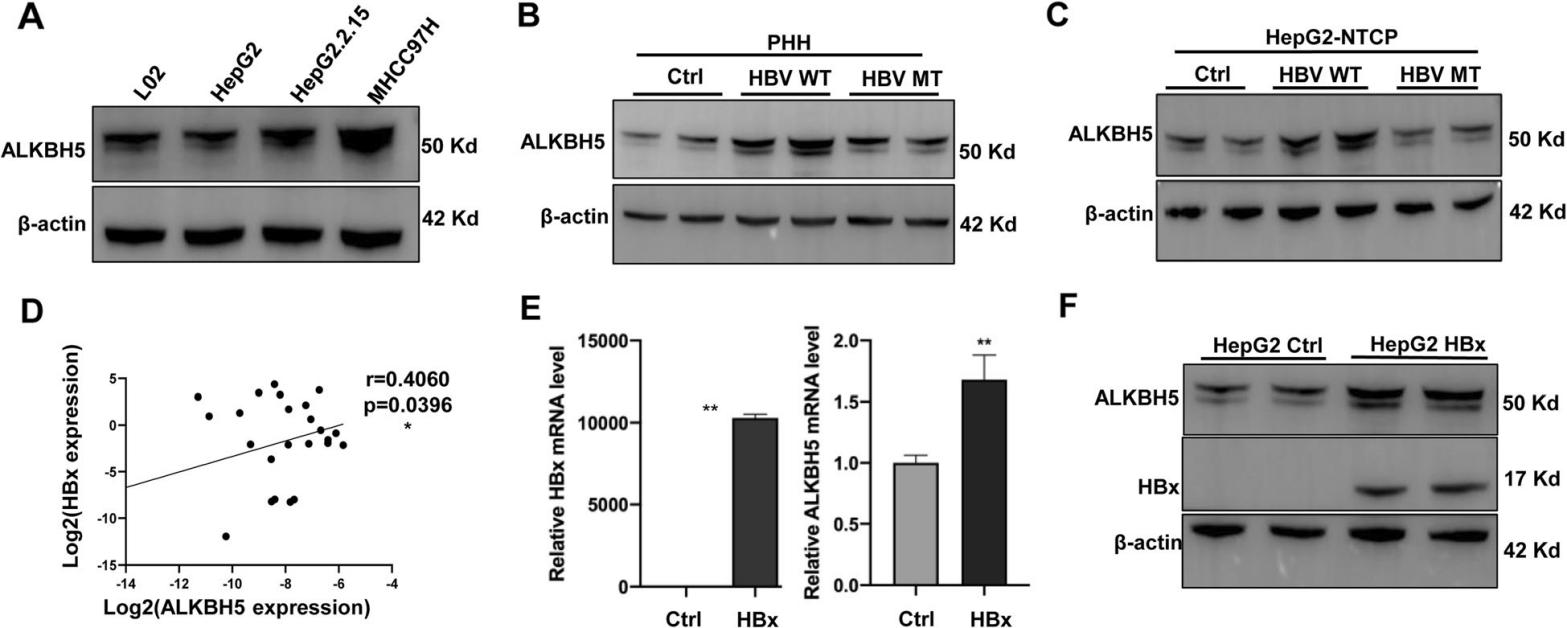
HBx up-regulates ALKBH5. **A** The protein level of ALKBH5 in the indicated cell lines. **B** ALKBH5 protein level in PHH cells with or without HBV WT or HBV MT virion infection. **C** ALKBH5 protein level in HepG2-NTCP cells with or without HBV WT or HBV MT virion infection. **D** Correlation analysis between HBx and ALKBH5 mRNA expression determined by RT-qPCR in tumors from 24 HBV-HCC patients. **E**, **F** ALKBH5 mRNA and protein levels in HepG2 cells with or without HBx transduction. WT, widetype; MT, mutant

As HBV could dysregulate host genes, we next explored whether HBV causes the increased ALKBH5 expression. Firstly, we checked ALKBH5 protein level in L02, HepG2, HepG2.2.15 and MHCC97H cell lines as the latter two cell lines contain HBV genome and produce HBV virus compared to the first two. Western blot indicated a higher ALKBH5 expression in HBV-infected cell lines (Fig. 3A) which implied the regulation relationship between HBV and ALKBH5. To directly assess the effects of HBV on ALKBH5 expression, we conducted in vitro HBV infection assay on PHH cells and HepG2-NTCP cells. Wild type HBV virion (HBV WT) could increase the ALKBH5 protein level while a mutation of HBx-depletion HBV virion (HBV MT) could not (Fig. 3B and C). HBx is not only vital to boost HBV transcription and replication, but also play critical roles in liver carcinogenesis by directly dysregulating host oncogenes and tumor suppressing genes. We further observed there were positive correlations between HBx and ALKBH5 in the HBV-HCC tissue (Fig. 3D), and we thus check whether overexpression of HBx promotes ALKBH5 expression. Indeed, HBx up-regulates both mRNA and protein level of ALKBH5 in HepG2 cells after HBx-overexpression plasmids transfection (Fig. 3E and F).

**Fig. 4:**
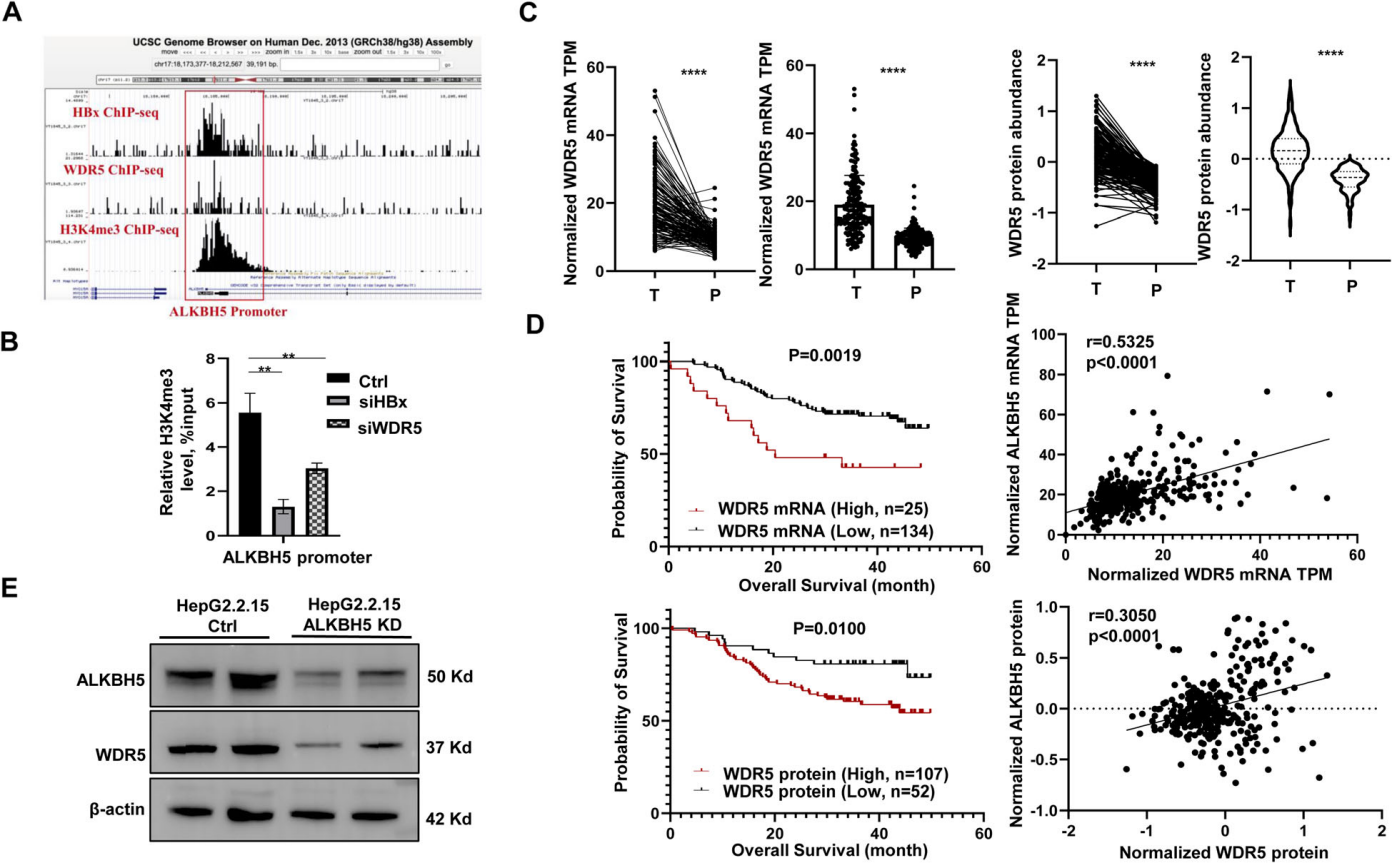
HBV up-regulates ALKBH5 via the HBx-WDR5-H3K4me3 axis. **A** HBx, WDR5 and H3K4me3 distribution at the *ALKBH5* gene locus. Snapshot generated by the UCSC Genome Browser. **B** The H3K4me3 level of *ALKBH5* promoter was measured by ChIP-qPCR after either HBx or WDR5 knockdown compared to the control. **C** WDR5 mRNA TPM score and protein abundance in tumor and paired peri-tumor from 159 Fudan HBV-HCC patients’ transcriptome and proteome dataset. **D** Left panel: Kaplan-Meier analysis of WDR5 mRNA TPM, protein abundance and overall survival of 159 Fudan HBV-HCC patients. Right panel: Correlation analysis between WDR5 and ALKBH5 mRNA TPM, protein abundance from 159 Fudan HBV-HCC patients’ transcriptome and proteome dataset. **E** WDR5 protein level in HepG2.2.15 cells with or without ALKBH5 knockdown. T, tumor; P, peri-tumor

Our previous study indicated that HBx hijacks WDR5 to promote target oncogenes’ promoter H3K4me3 modification [6], which suggests that the increased ALKBH5 expression in HBV-HCC might be mediated through the HBx-WDR5-H3K4me3 axis. After re-analyzing the HBx, WDR5 and H3K4me3 ChIP-seq data in our previous study, we found that HBx and WDR5 binds to the promoter of ALKBH5 gene with the H3K4me3 modification as shown in the UCSC genome browser (Fig. 4A). Furthermore, knocking down either HBx or WDR5 by small interfering RNA transfection decreased H3K4me3 modification level of ALKBH5 gene’s promoter as evidenced by ChIP-qPCR (Fig. 4B). We further data mined 159 Fudan HBV-HCC cohort to analyze the correlation between WDR5 and ALKBH5 expression, we found that WDR5 was indeed increased in the HBV-HCC at both mRNA and protein level (Fig. 4C) and the increased WDR5 level predicted a poor prognosis in accordance with previous reports [6] (Fig. 4D, Left panel). Moreover, the expression of WDR5 were positively related to the expression of ALKBH5 in the 159 Fudan HBV-HCC cohort (Fig. 4D, Right panel). The relationship between WDR5 and ALKBH5 was further defined by knockdown of ALKBH5 in HepG2.2.15 cells, and we observed that the down-regulated expression of WDR5 after ALKBH5 knockdown (Fig. 4E). These results support the notion that HBV up-regulates ALKBH5 via the HBx-WDR5-H3K4me3 axis.

### ALKBH5 promotes the growth and migration of hepatoma cells

**Fig. 5:**
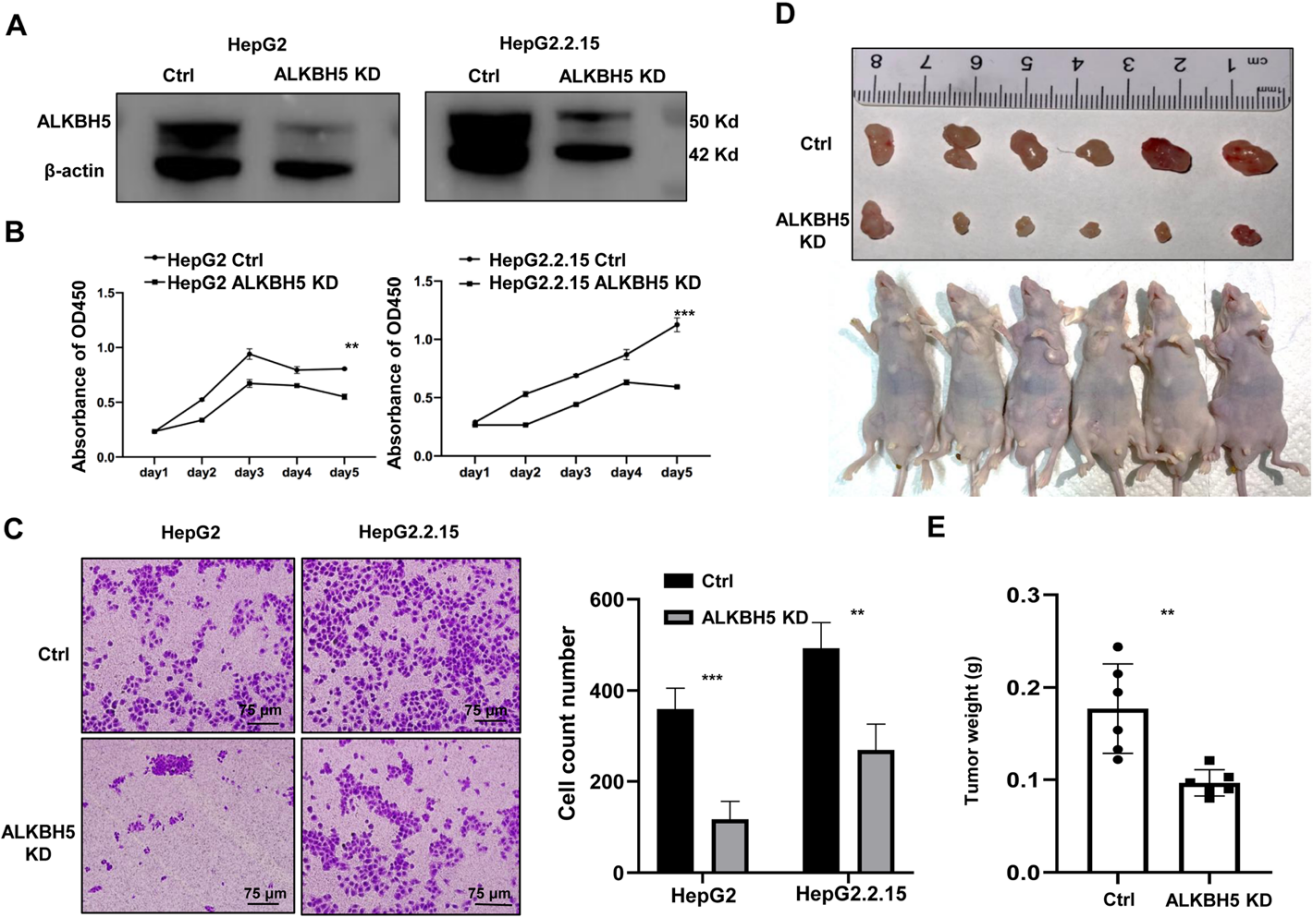
ALKBH5 promotes the growth and migration of hepatoma cells. **A** Efficiency of ALKBH5 knockdown (KD) in HepG2 and HepG2.2.15 cells were evaluated by Western blot assay. **B** Proliferation rates of HepG2 and HepG2.2.15 with or without silencing of ALKBH5 were detected by CCK8 assay. **C** Effect of knockdown of ALKBH5 on migration rates of HepG2 and HepG2.2.15 were detected by transwell assay. **D**, **E** Tumor size and weight in each group. 1 × 10^7^ of HepG2.2.15 cells (control) or HepG2.2.15 cells with ALKBH5-knockdown (KD) were subcutaneously injected into nude mice, and the tumor size (**D**) and weight (**E**) were measured 3 weeks after tumor cells inoculation

As ALKBH5 is highly expressed in HBV-HCC, we wondered the tumorigenic function of ALKBH5 in HBV-induced carcinogenesis. Silencing ALKBH5 inhibited proliferation rates of both HepG2 and HepG2.2.15 cells (Fig. 5A and B). Correspondingly, transwell assay showed that ALKBH5 supported tumor’s migration ability of both HepG2 and HepG2.2.15 cells (Fig. 5C). These silencing results indicated that ALKBH5 functions as the oncogene in HBV-independent manner. The role of ALKBH5 in HCC was further addressed in a nude mouse model. When HepG2.2.15 cells with stable ALKBH5 knockdown were transferred to the subcutaneous of nude mice, the sizes of xenografted tumors and the weights decreased than the control group (Fig. 5D and E). These results illustrated the tumor-promoting activity of HBV might be mediated, at least partially, by the ALKBH5 in HBV-HCC cells.

### ALKBH5 favors HBx mRNA stability by regulating its m^6^A modification

**Fig. 6:**
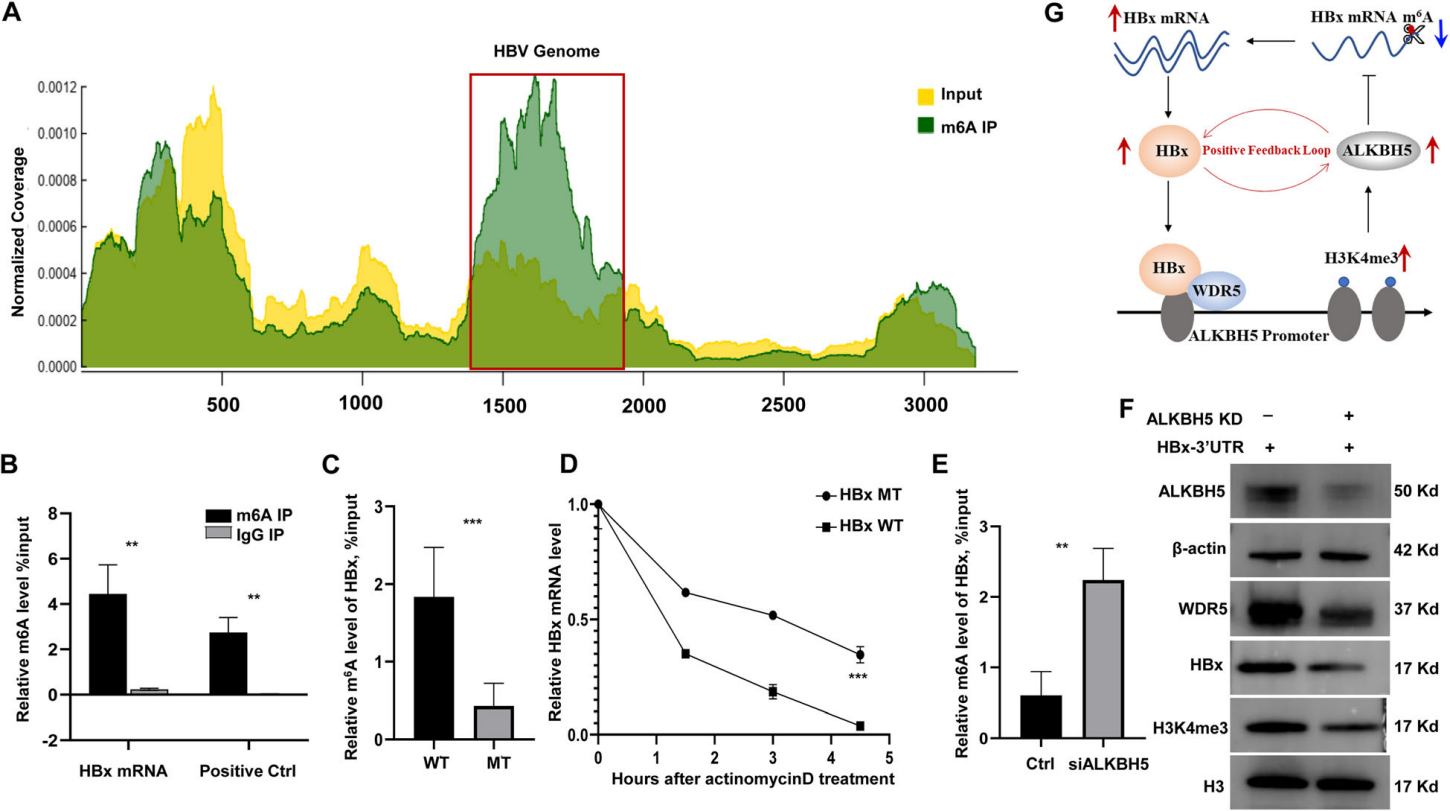
ALKBH5 favors HBx mRNA stability by regulating its m^6^A modification. **A** Map of m^6^A modification sites in the HBV *ayw* genome by MeRIP-seq of polyA-RNA isolated from HepG2.2.15 cells. Read coverage, normalized to the total number of reads mapping to the viral genome, is in green for MeRIP-seq and in yellow for input RNA-seq. The major m^6^A peak on HBV genome in the red box. **B** MeRIP-qPCR analysis of HBx mRNA, compared to the positive control. **C** MeRIP-qPCR analysis of HBx 3’UTR WT and MT mRNA. **D** RNA decay assay to detect the half-life of HBx 3’UTR WT and MT mRNA. HepG2 cells were transfected with HBx 3’UTR WT or MT plasmids and RT-qPCR was performed after actinomycin D was added. **E** MeRIP-qPCR analysis of HBx mRNA after silencing of ALKBH5. **F** Western blot analysis of HBx, WDR5 and H3K4me3 protein after silencing of ALKBH5. **G** Cartoon illustration of the positive feedback loop of HBx-ALKBH5.WT, widetype; MT, mutant

A recently published study reported that HBV encodes RNA including pre-genomic RNA with m^6^A modification at the 3’UTR and 5′ UTR, and the m^6^A modification of 3’UTR affects the HBV RNA stability in HBV expressing HepG2 cells [15]. Therefore, the increased ALKBH5 in HepG2.2.15 cells of the present study probably involve in epitranscriptomics regulation of HBV RNA. We thus use MeRIP-seq for HepG2.2.15 to detect whether there are m^6^A modification on HBV RNA in our study. We finally detected one major m^6^A peak on HBV RNA, similar with the previous report [15] (Fig. 6A). As HBx is the determinant of both HBV replication and HBV-induced carcinogenesis, we focused on the m^6^A modification on HBx 3’UTR. Firstly, we constructed both wildtype and A1907C mutation of HBx 3’UTR overexpression plasmids, and transfected the plasmids into HepG2 cells. MeRIP-qPCR validated that there was m^6^A modification on HBx 3’UTR WT mRNA compared to the positive control (Fig. 6B). Furthermore, the m^6^A modification level was dramatically decreased at the HBx 3’UTR MT mRNA than the WT (Fig. 6C). We also noticed that HBx 3’UTR MT mRNA processed more stability than the WT by RNA decay assay (Fig. 6D), which indicates that m^6^A modification on HBx 3’UTR promotes its mRNA decay. To further directly determine whether the ALKBH5 affect the m^6^A modification of HBx mRNA, we knocked down ALKBH5 in the HepG2-HBx 3’UTR WT cells, and the MeRIP-qPCR assay indicated that ALKBH5 silencing induced a higher m^6^A modification of HBx mRNA (Fig. 6E), in accordance with the decreased protein level of HBx (Fig. 6F), which suggests that ALKBH5 could regulate HBx expression level by modifying m^6^A level of HBx mRNA. In addition, we also conducted western blot assay for WDR5 and H3K4me3 with or without knockdown of *ALKBH5* gene (Fig. 6F), and we found that silencing of ALKBH5 decreased not only HBx protein but also WDR5 and H3K4me3 level, which indicated that ALKBH5 might participate in the epigenetic regulation of HBx-WDR5-H3K4me3 axis [6].

Therefore, we verified that HBx up-regulates ALKBH5 by the histone modification of H3K4me3 while ALKBH5 increases HBx via the mRNA modification of m^6^A, which may form a positive loop between HBx and ALKBH5 (Fig. 6G).

## Discussion

HBV exerts its tumor-promoting function by inducing abnormal epigenetics regulation of host cells, however, whether the newly defined epitranscriptomics regulation involves in HBV-induced epigenetics instability is limited studied. In the present study, we found ALKBH5, one of m^6^A epitranscriptomics enzyme, was highly increased in HBV-HCC tissues with prognosis prediction value, and targeting ALKBH5 inhibits tumor cells’ growth and migration. Furthermore, HBV up-regulates ALKBH5 expression through HBx-WDR5-H3K4me3 axis, and ALKBH5 promotes HBx mRNA stability by decreasing m^6^A modification, thus forming a positive feedback loop.

Kim et al. have reported that HBV increases the m^6^A writers METTL3/14 expression and thus decreases PTEN expression by regulating m^6^A modification of cognate RNAs [14]. However, further study is needed to clarify more epitranscriptomics regulation of HBV on host cell including whether and how HBV affects other m^6^A enzymes expression. In present study, we find ALKBH5 is increased by HBV infection, providing new hints that epitranscriptomics regulation is involved in HBV-induced HCC by altering the expression of m^6^A enzyme ALKBH5. Nevertheless, further studies are needed to profile the down-stream target genes with abnormal m^6^A modification by the HBx-ALKBH5 loop, which would provide more potential targets for HBV-related HCC treatment.

ALKBH5 has been reported to be dysregulated with either tumor-promoting or tumor-inhibiting roles in various cancer types, including pancreatic cancer, lung cancer, breast cancer and HCC [26]. The diverse roles played by ALKBH5 might be dependent on the tumor context. Regarding to HCC, previous studies reported that ALKBH5 is decreased and thus suppresses malignancy in HCC [22]. However, in our present study, including total 99 cases of clinical samples and combining with further data by mining the database from two cohorts of 159 Fudan Chinese HBV-HCC and 115 TCGA HBV-HCC patients [24, 27], we confirmed that ALKBH5 is up-regulated in most HBV-HCC tissues and predicts a poor prognosis. Functionally, the proliferation and migration arrays in vitro and in vivo suggest the tumor promoting role of the highly expressed ALKBH5 in HCC. Thus, the present data provide a further understanding of ALKBH5 expression pattern and biological function in HCC.

As for the controversies of ALKBH5 expression in HCC tissues in the published literature, we think it may be due to the heterogeneous HCC background. The published reports do not describe whether those HCC tissues are HBV-related or not. In the present study, we only included the HBV-related HCC tissues to evaluate the role of ALKBH5, which might be a possible reason for the controversy of ALKBH5 expression in HCC tissues between our study and the published works. On the other hand, there are diverse molecular subtypes of HBV-HCC, which might determine the different expression pattern for certain genes [24, 27]. In addition, the effects of ALKBH5 on carcinogenesis might relate to the stage of tumor and the different downstream molecules as shown by other epitranscriptomics enzyme such as FTO [28, 29].

In conclusion, our study reports the highly expressed ALKBH5 induced by HBV plays a critical role in HCC malignancy by forming a positive feedback loop between HBx and ALKBH5, which could be a potential prognostic indicator and a potential novel therapeutic target for HBV-HCC.

## Supplementary Information

**Additional file 1.**

## Data Availability

RNA-seq and Mass spectrum (MS) data of 159 liver samples (paired tumor and peri-tumor) from CHCC-HBV patients are available downloaded through the URL: https://www.biosino.org/node/project/detail/ OEP000321. RNA-seq data of 115 HBV-HCC samples from TCGA were downloaded from the URL: https://cbioportal-datahub.s3.amazonaws.com/lihc_tcga.tar.gz and the HBV infection and genotype information were downloaded from the URL: https://ars.els-cdn.com/content/image/1-s2.0-S2589004221003369-mmc2.zip. MeRIP-seq and input RNA datasets generated and analysed during the current study are not publicly available due other relevant study is still in progress but are available from the corresponding author on reasonable request.

## Abbreviations

HBx: Hepatitis B virus encode X protein
HCC: Hepatocellular carcinoma
ALKBH5: Alkb homolog 5
m^6^A: N6-methyladenosine
WDR5: WD repeat domain 5
H3K4me3: Histone 3 lysine 4 trimethylation
NTCP: Na+/ taurocholate cotransporting polypeptide
RT-qPCR: Quantitative reverse transcription PCR
CHIP-qPCR: Chromatin Immunoprecipitation quantitative PCR
MeRIP-qPCR: Methylated RNA quantitive PCR
siRNA: Small-interfering RNA
IP: Immunoprecipitation
