# Supplementary material for: A positive-feedback loop between HBx and ALKBH5 promotes hepatocellular carcinogenesis
Source: BMC Cancer. 2021 Jun 10;21:686. doi: 10.1186/s12885-021-08449-5 (PMC8194239; doi:10.1186/s12885-021-08449-5)
Supplement: Supplementary file 1 — Additional file 1. [file 12885_2021_8449_MOESM1_ESM.pdf]

Figure 1B

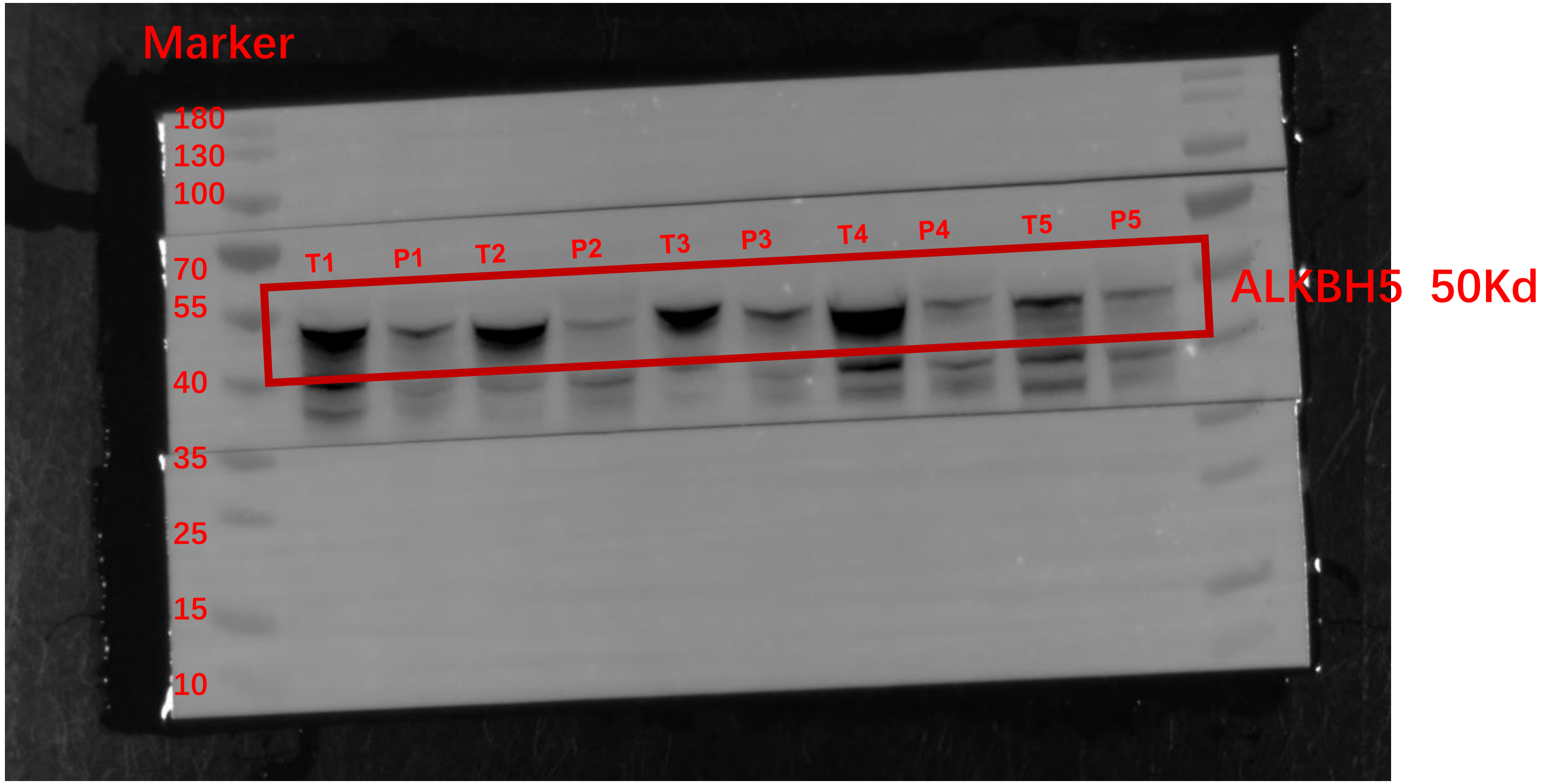

Marker
180
130
100
70
55
40
35
25
15
10
T1
P1
T2
P2
T3
P3
T4
P4
T5
P5
ALKBH5 50Kd

Figure 1B

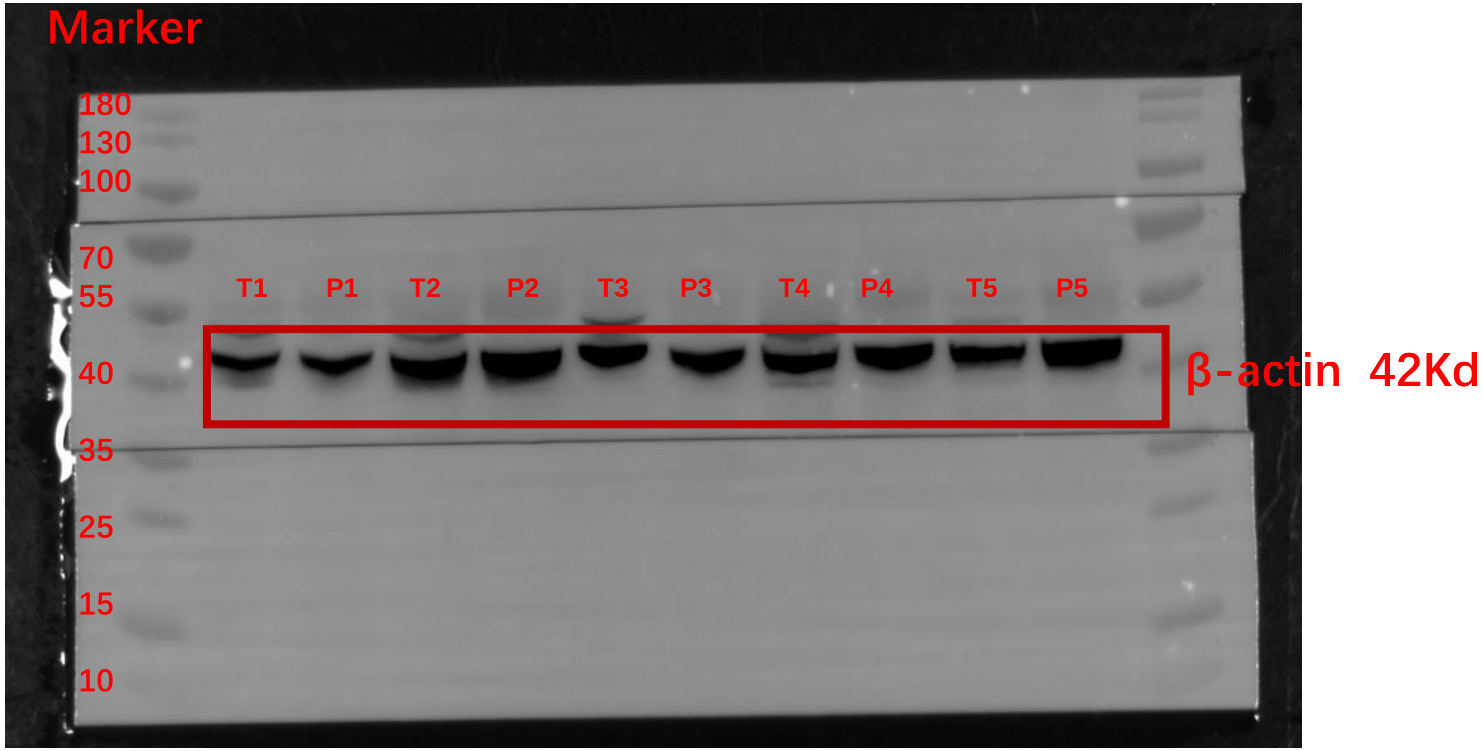
Marker
180
130
100
70
55
40
35
25
15
10
T1
P1
T2
P2
T3
P3
T4
P4
T5
P5
β-actin 42Kd

Figure 1B

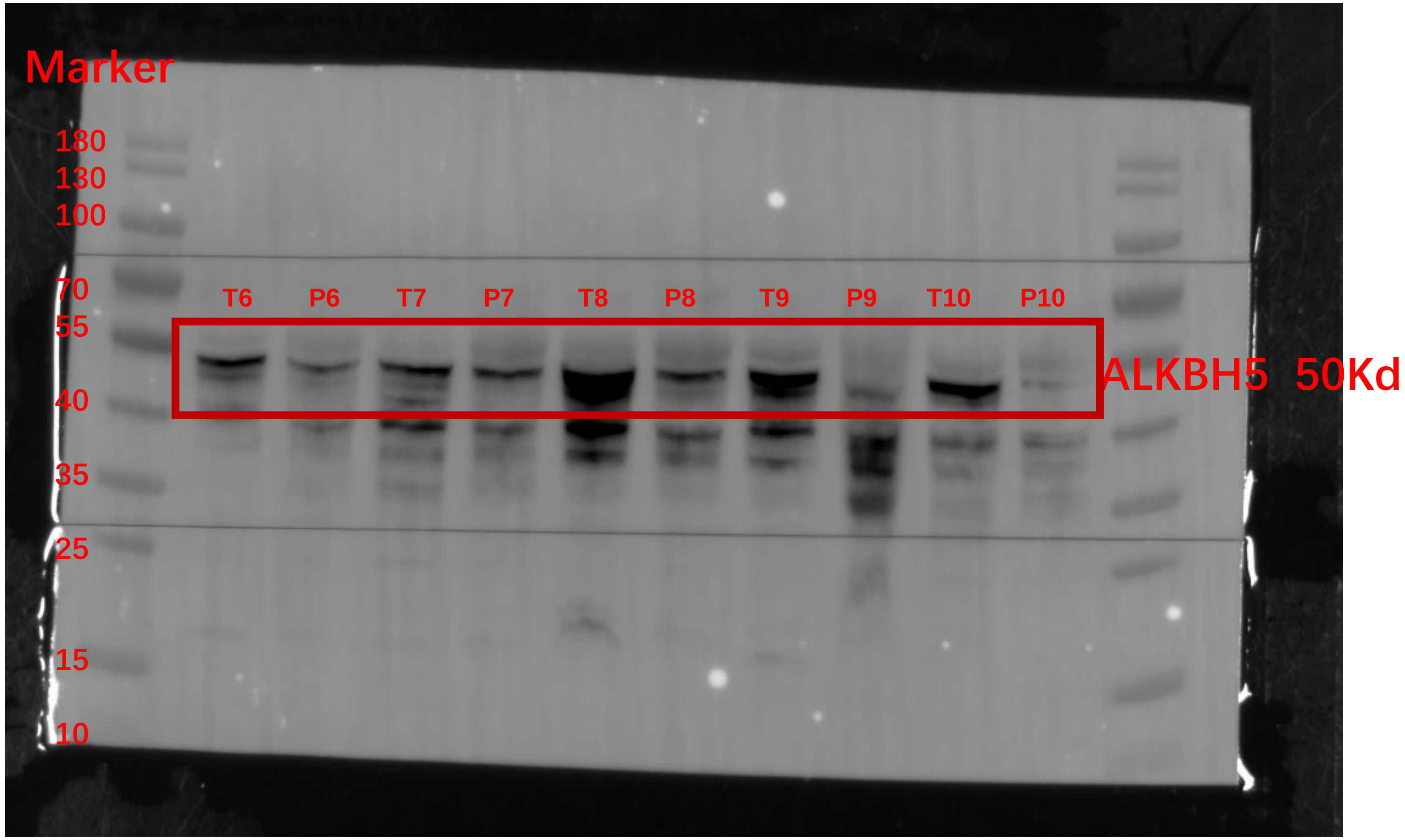
Marker
180
130
100
70
55
40
35
25
15
10
T6
P6
T7
P7
T8
P8
T9
P9
T10
P10
ALKBH5 50Kd

Figure 1B

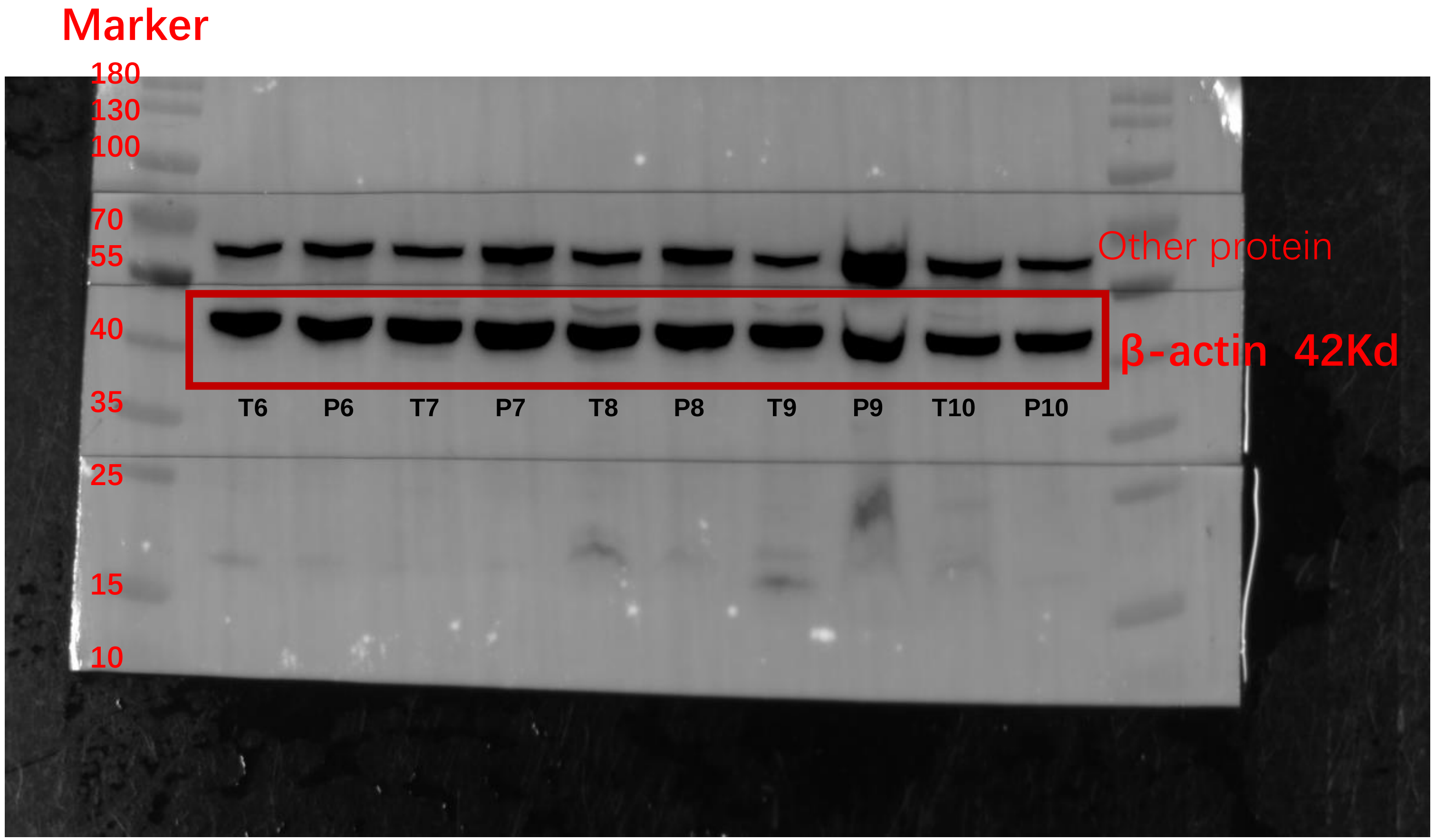
Marker
180
130
100
70
55
40
35
25
15
10
Other protein
β-actin 42Kd
T6
P6
T7
P7
T8
P8
T9
P9
T10
P10

Figure 1B

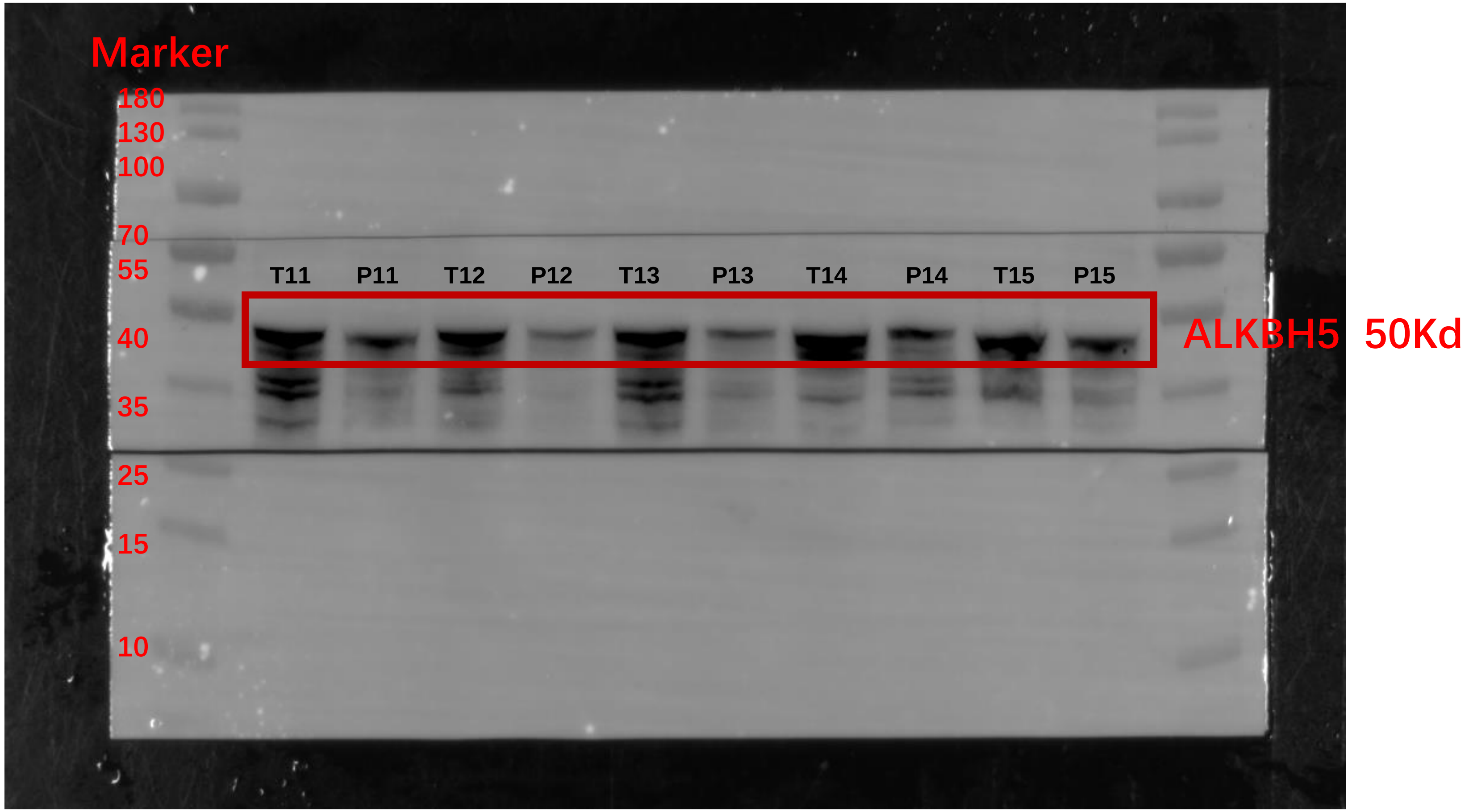

Marker
180
130
100
70
55
40
35
25
15
10
T11
P11
T12
P12
T13
P13
T14
P14
T15
P15
ALKBH5 50Kd

Figure 1B

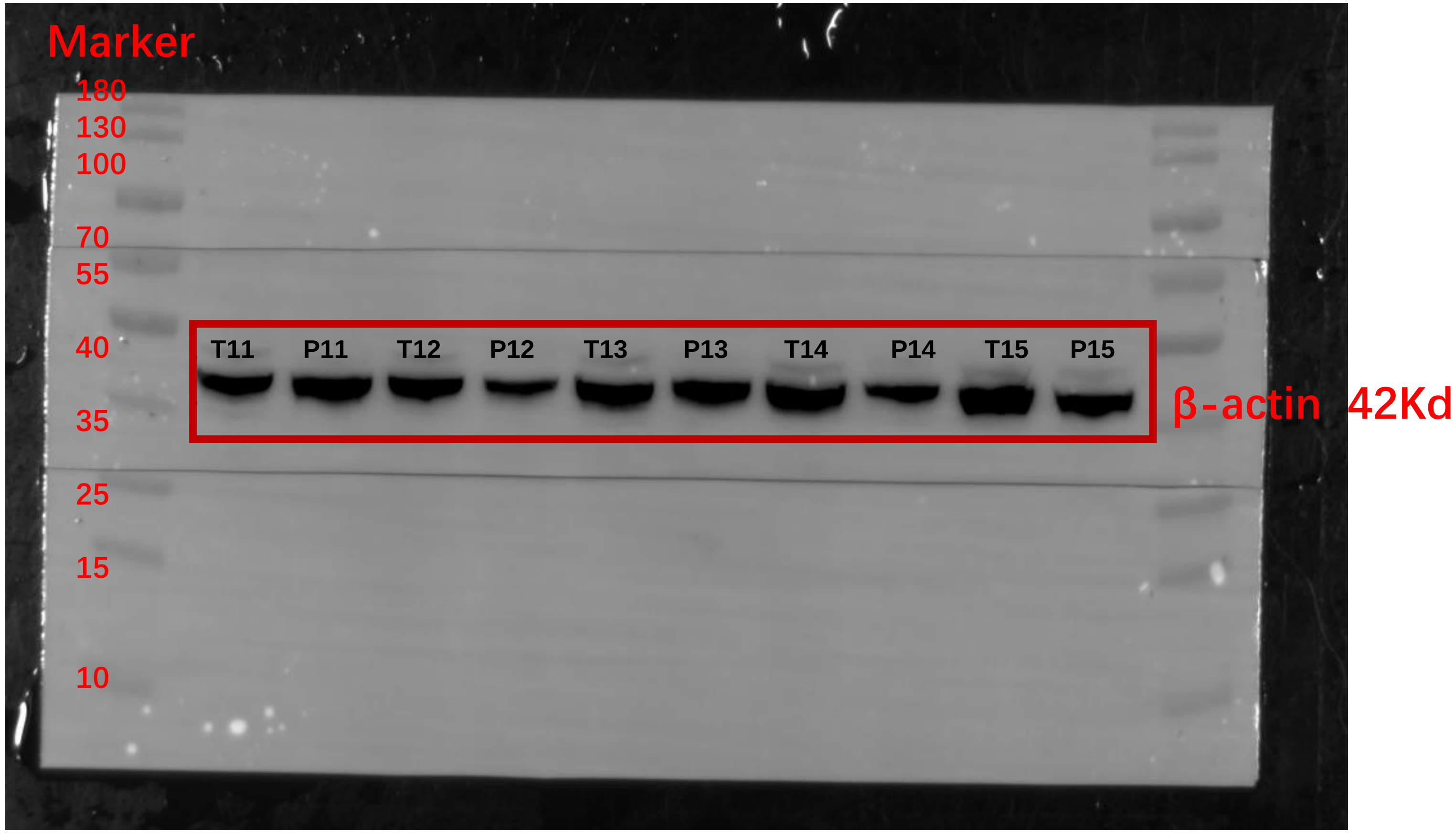
Marker
180
130
100
70
55
40
35
25
15
10
T11
P11
T12
P12
T13
P13
T14
P14
T15
P15
β-actin 42Kd

Figure 1B

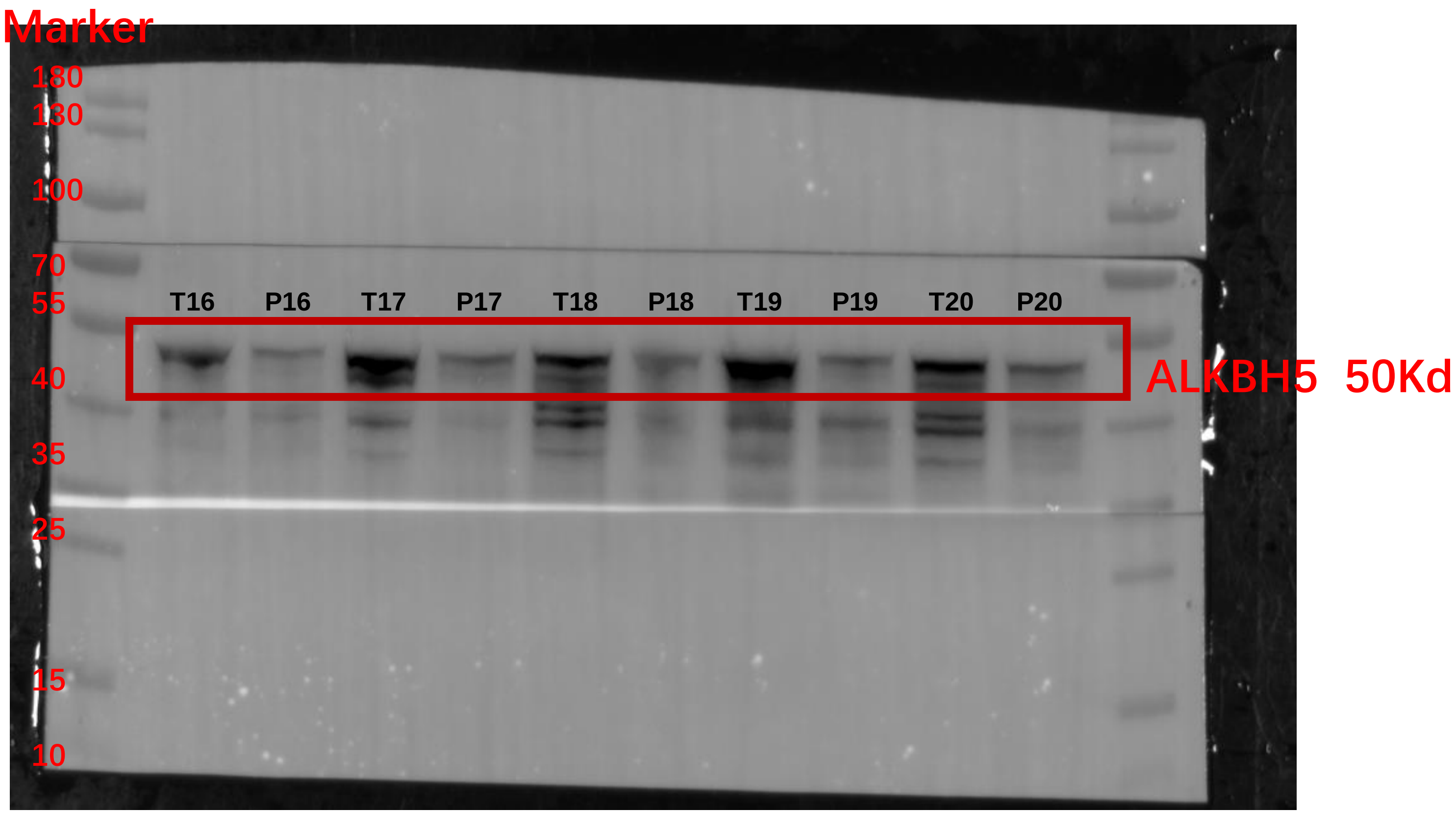
Marker
180
130
100
70
55
40
35
25
15
10
T16
P16
T17
P17
T18
P18
T19
P19
T20
P20
ALKBH5 50Kd

Figure 1B

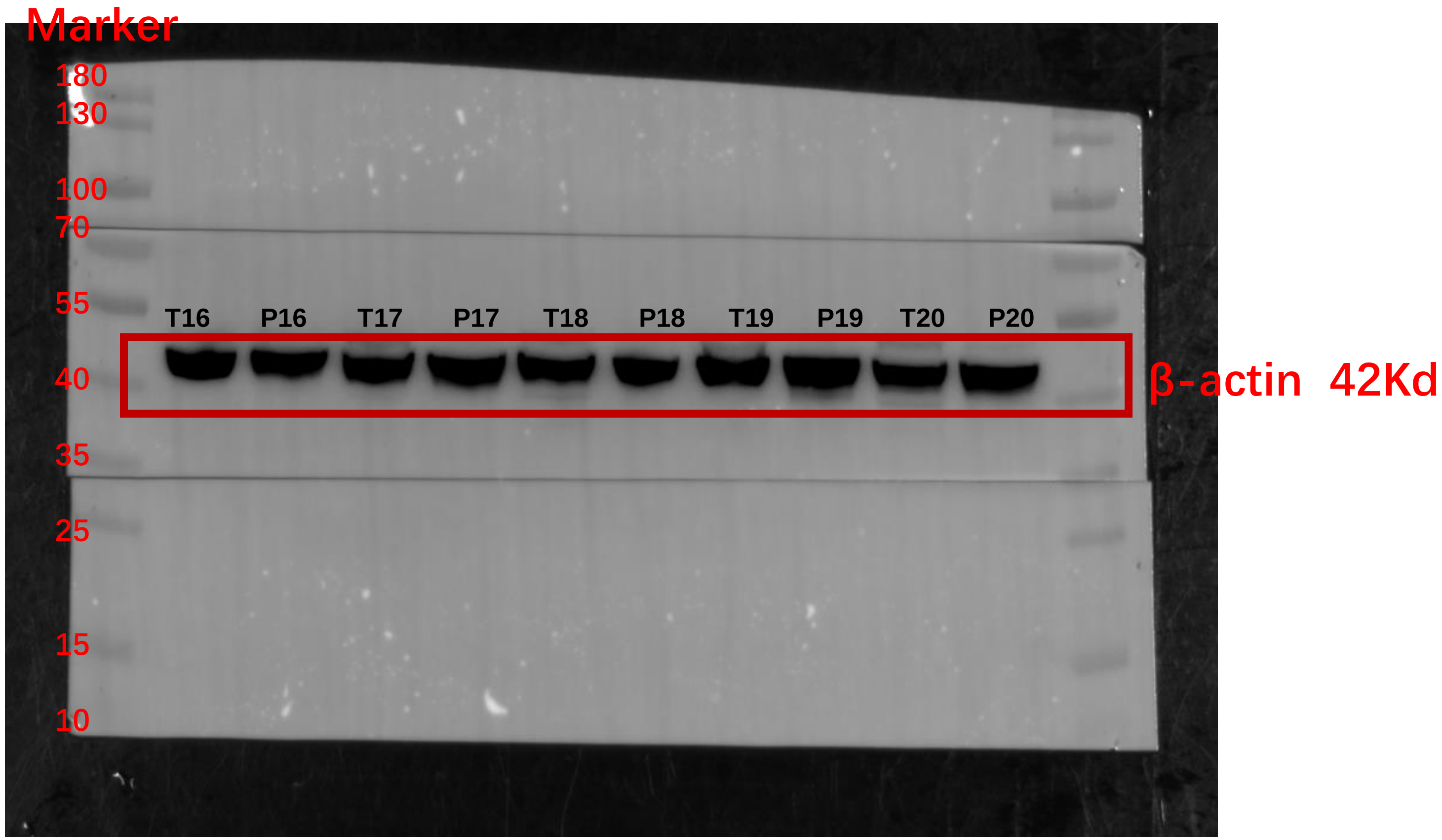
Marker
180
130
100
70
55
40
35
25
15
10
T16
P16
T17
P17
T18
P18
T19
P19
T20
P20
β-actin 42Kd

Figure 3A

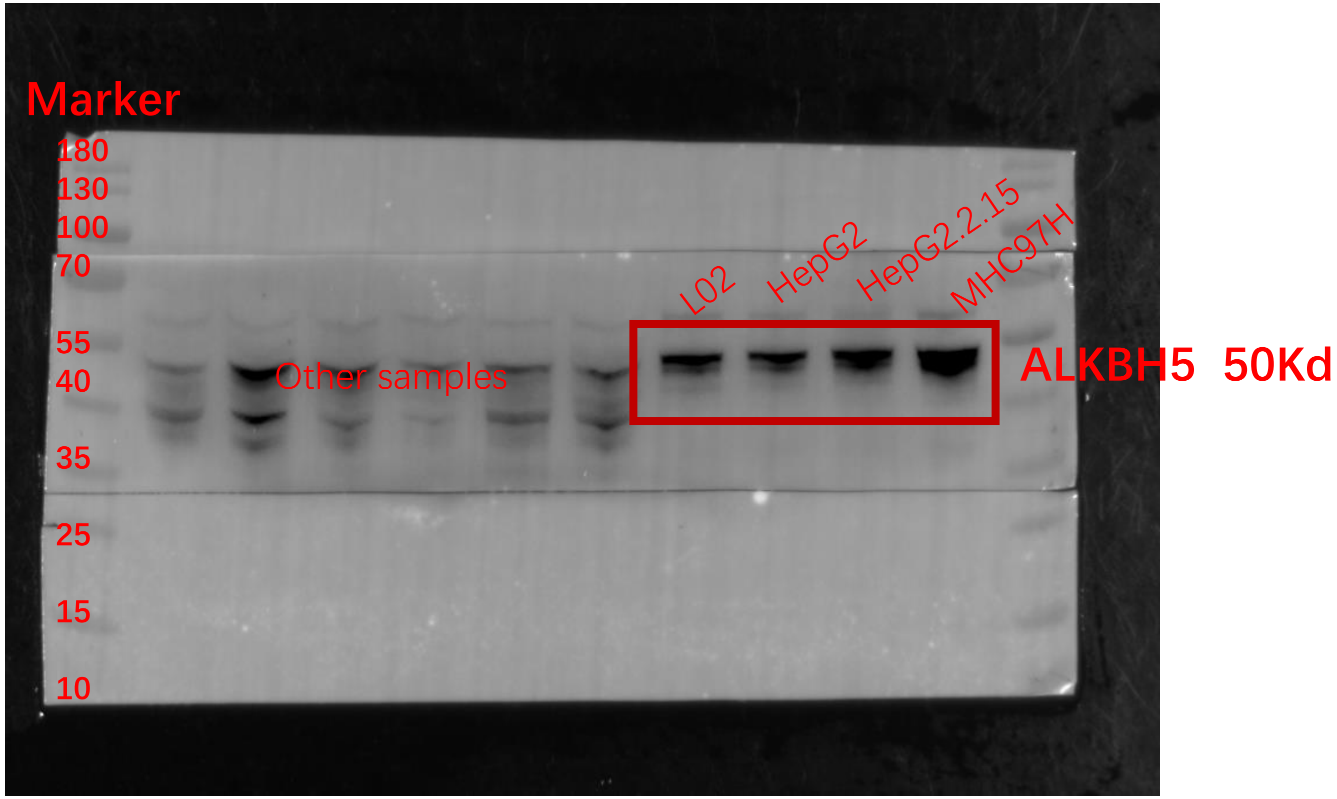
Marker
180
130
100
70
55
40
35
25
15
10
Other samples
L02
HepG2
HepG2.2.15
MHC97H
ALKBH5 50Kd

Figure 3A

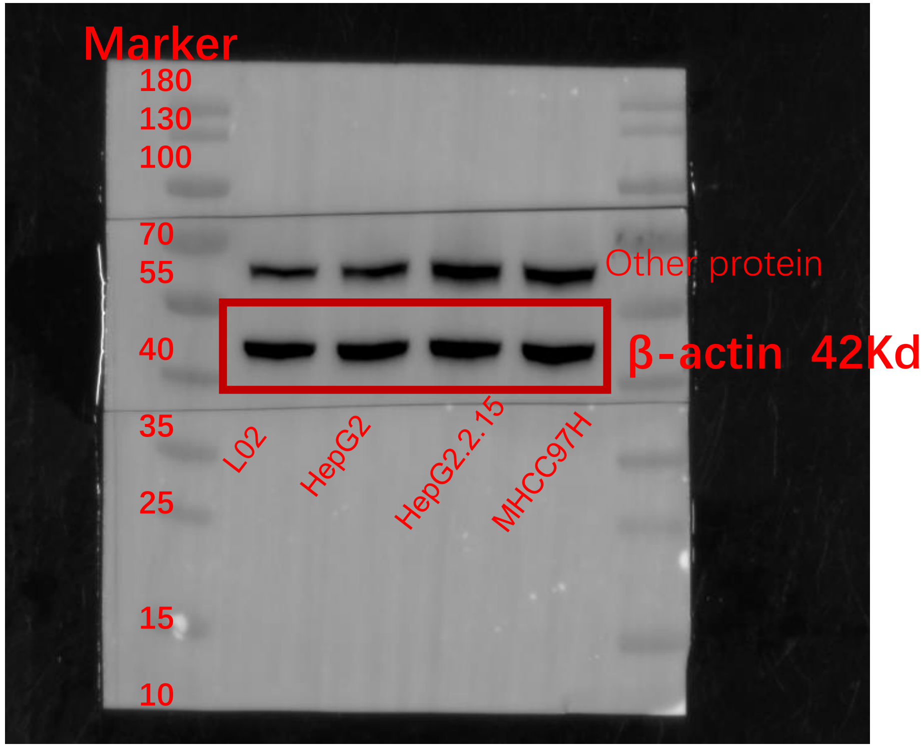

Marker
180
130
100
70
55
40
35
25
15
10
Other protein
β-actin 42Kd
L02
HepG2
HepG2.2.15
MHCC97H

Figure 3B

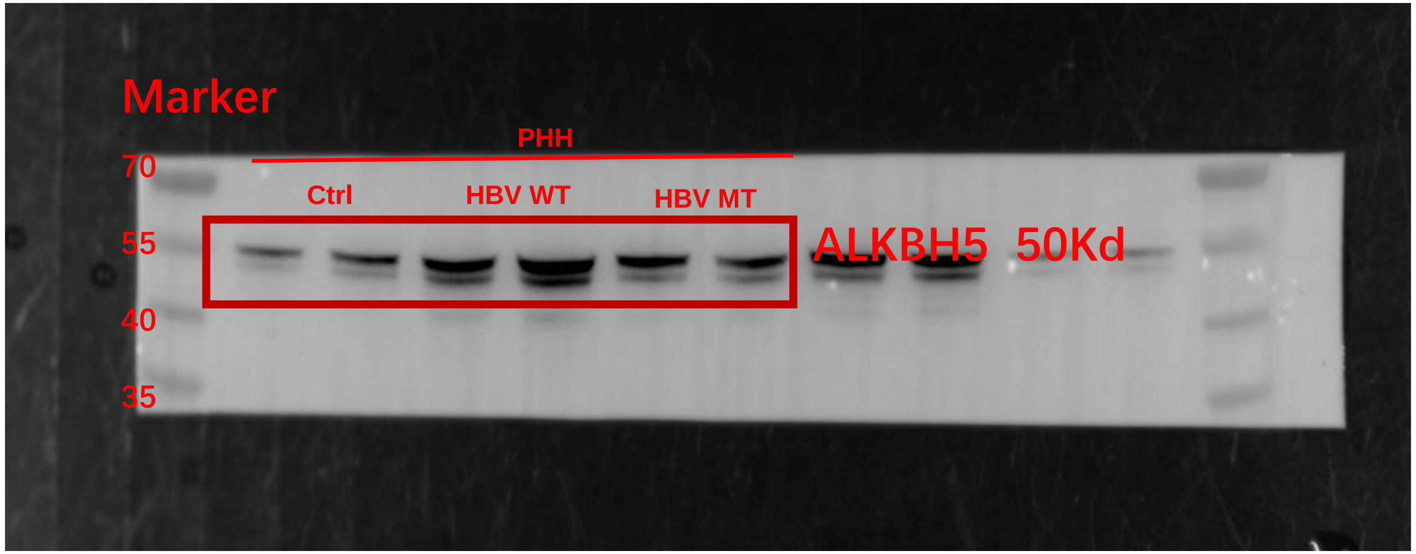

Figure 3B

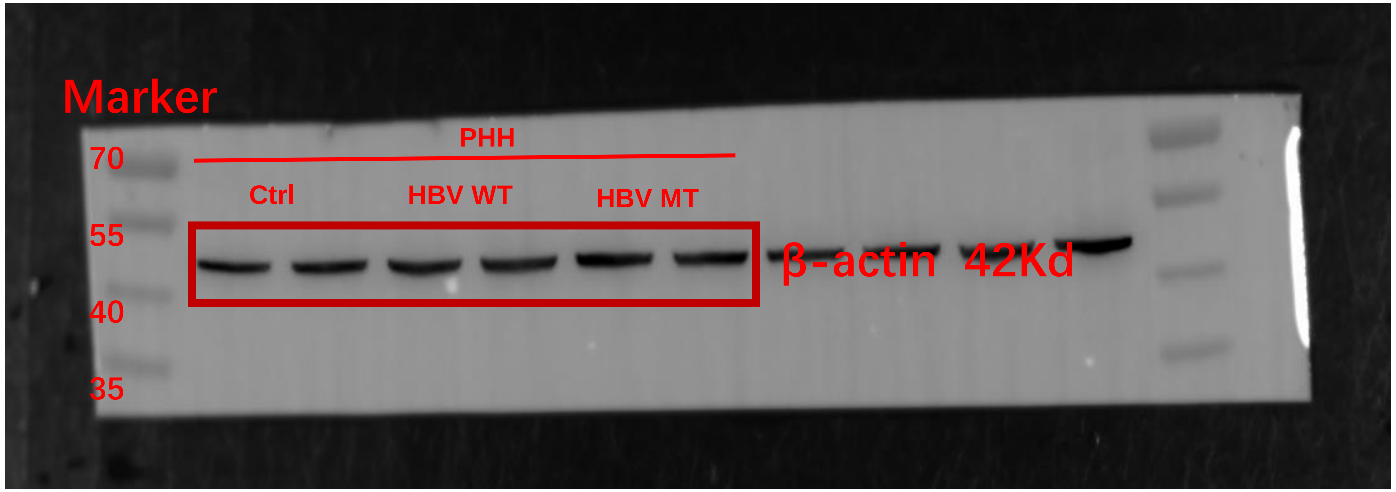

Figure 3C

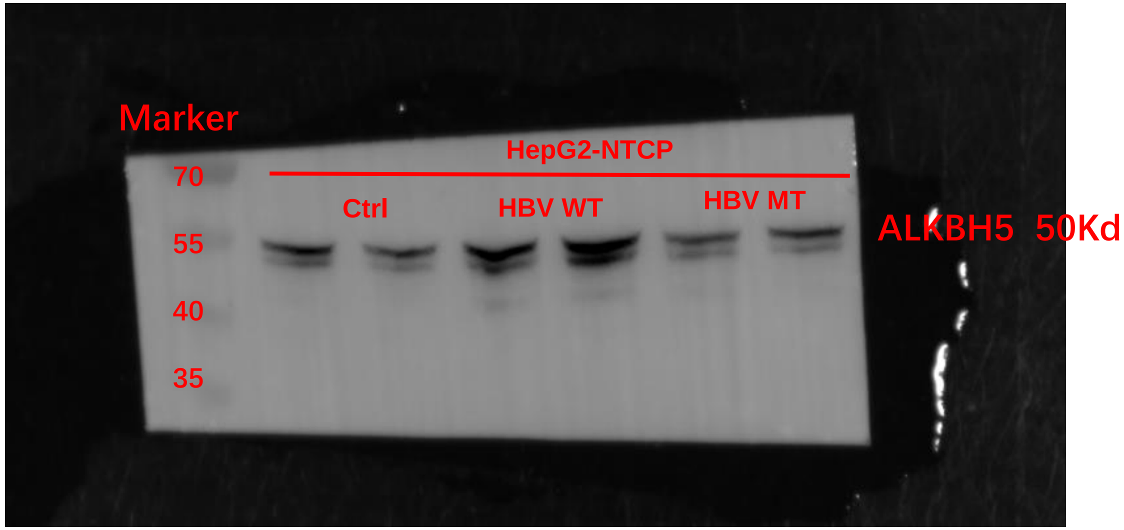

Figure 3C

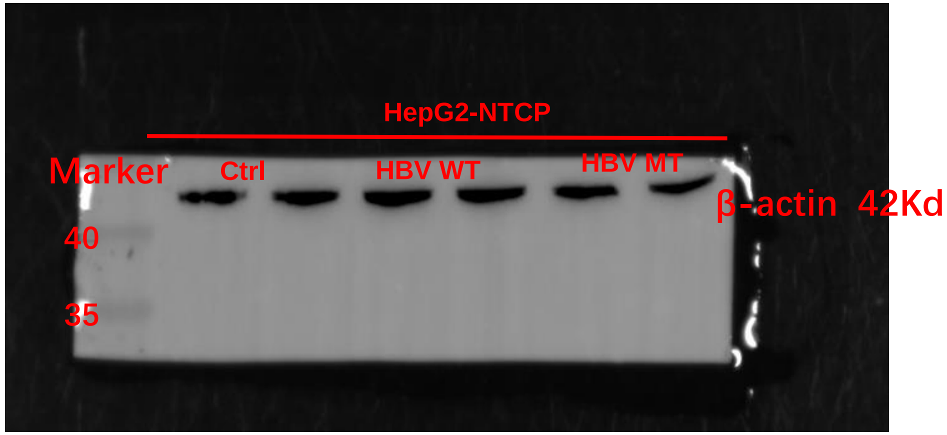

Figure 3F

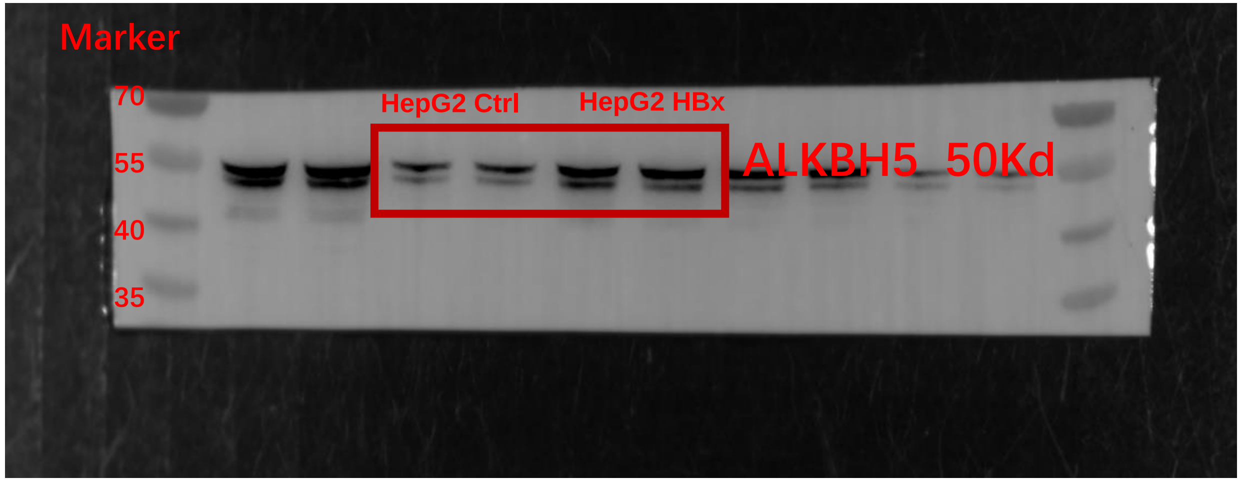

Figure 3F

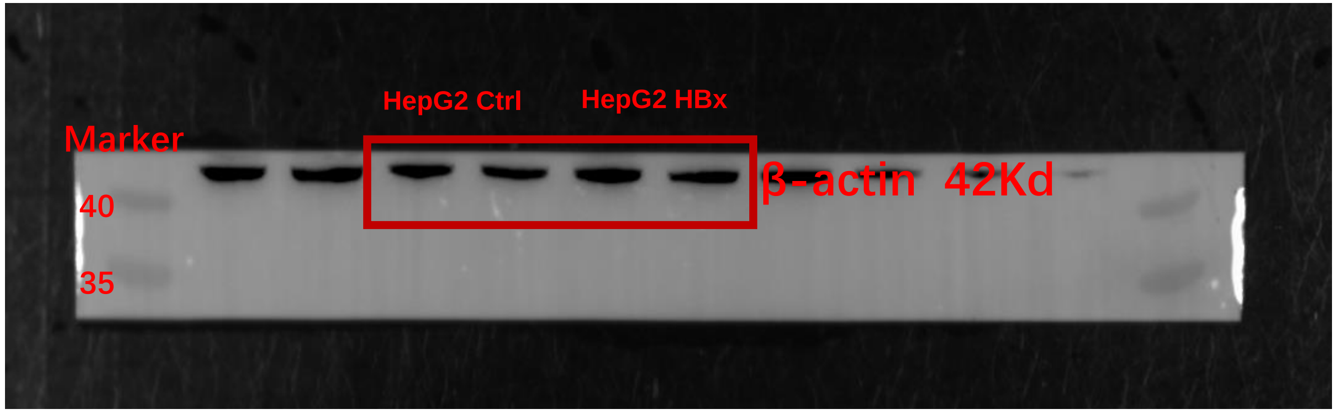

Figure 3F

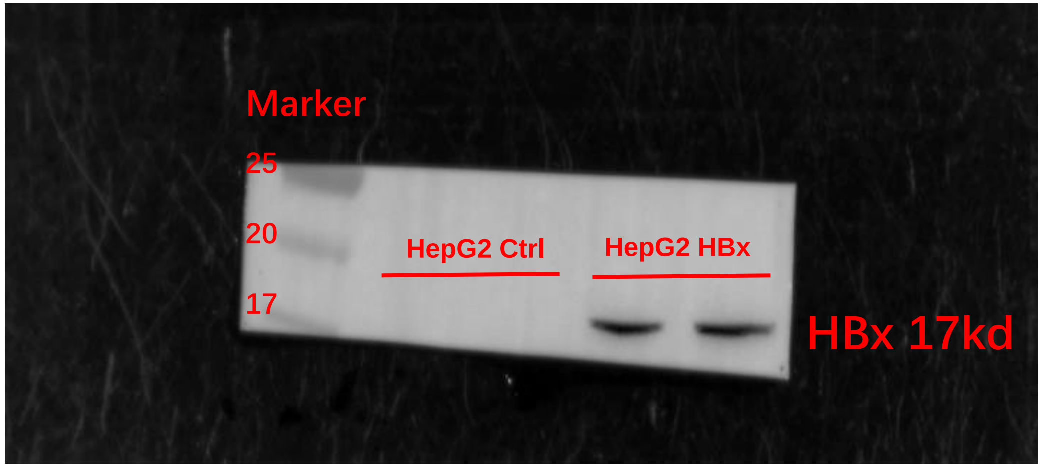

Figure 4E

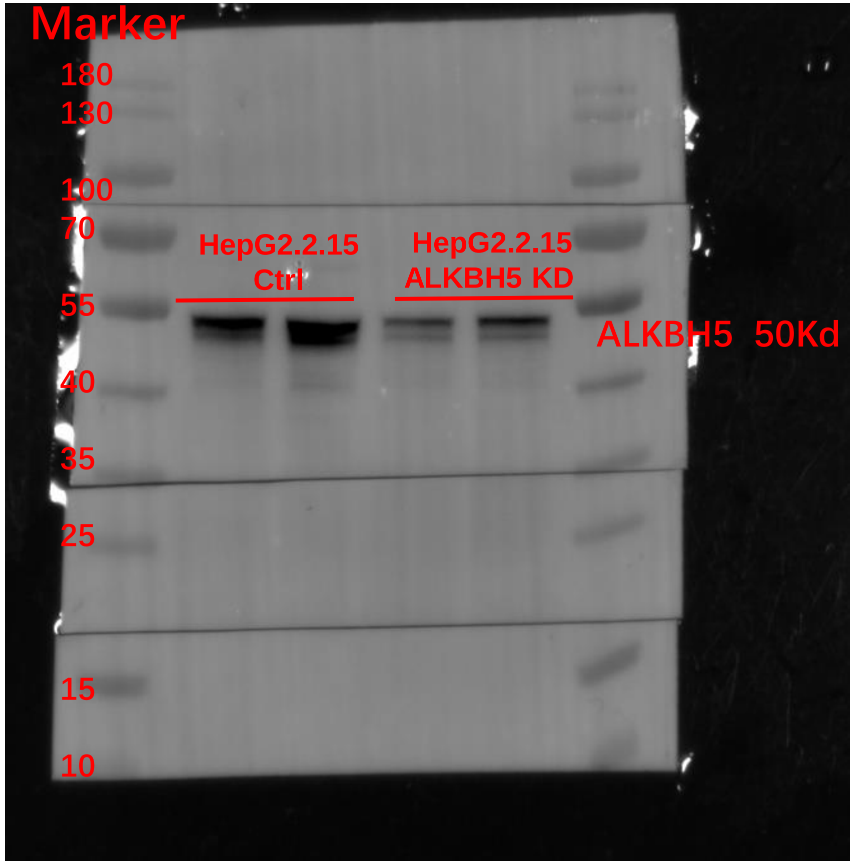
Marker
180
130
100
70
55
40
35
25
15
10
HepG2.2.15
Ctrl
HepG2.2.15
ALKBH5 KD
ALKBH5 50Kd

Figure 4E

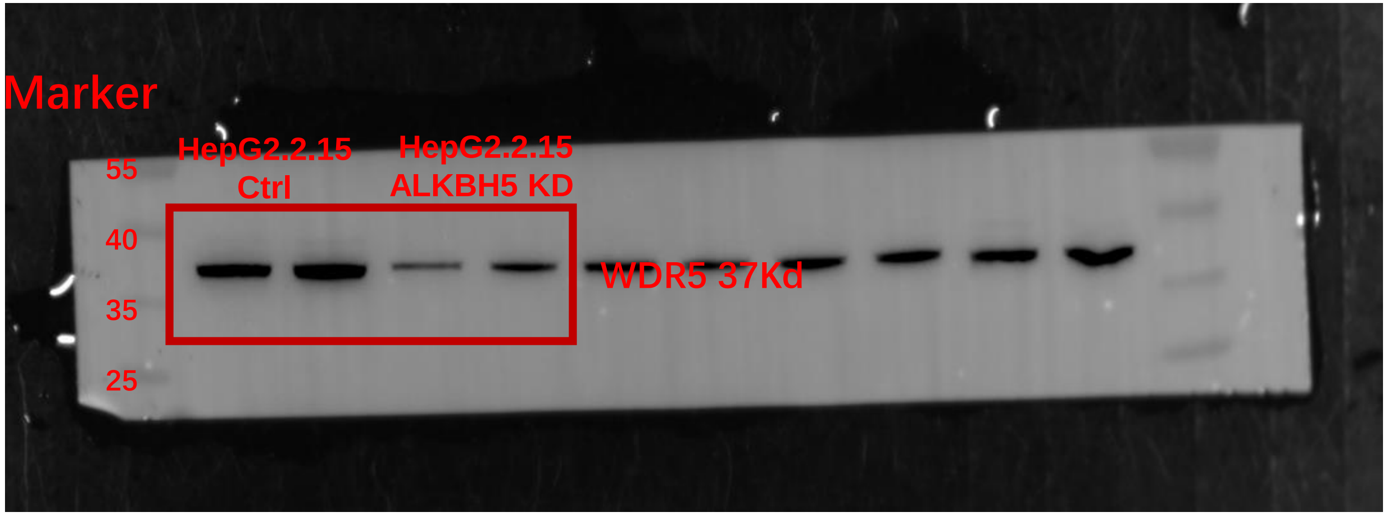

Marker
HepG2.2.15
Ctrl
HepG2.2.15
ALKBH5 KD
55
40
35
25
WDR5 37Kd

Figure 4E

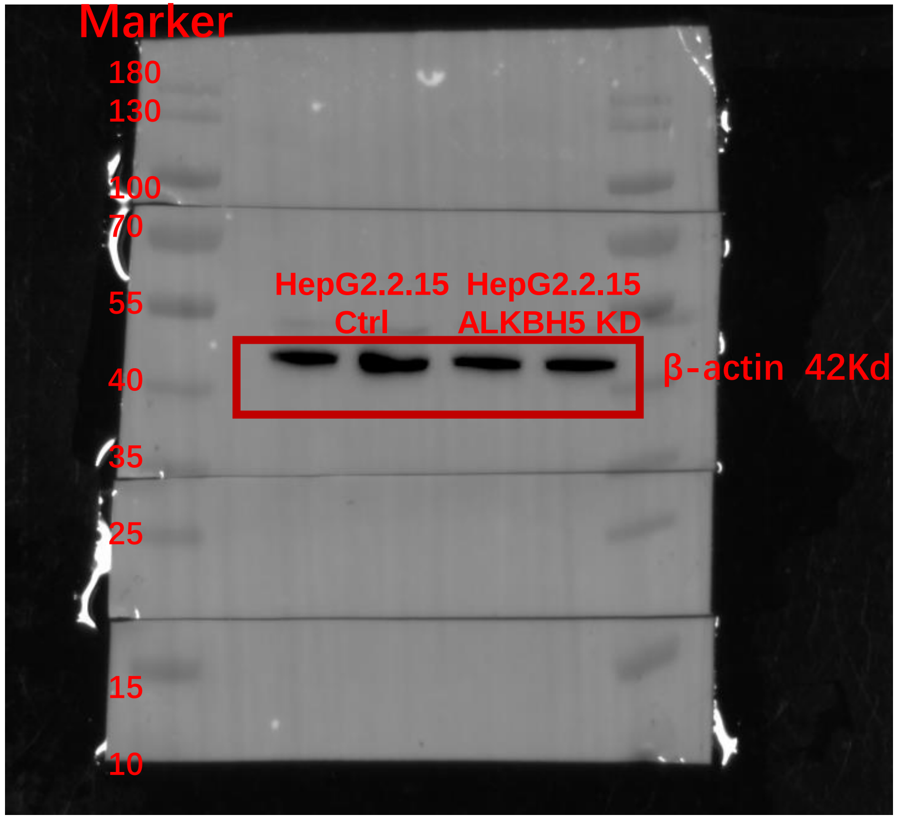

Marker
180
130
100
70
55
40
35
25
15
10
HepG2.2.15 Ctrl
HepG2.2.15 ALKBH5 KD
β-actin 42Kd

Figure 5A

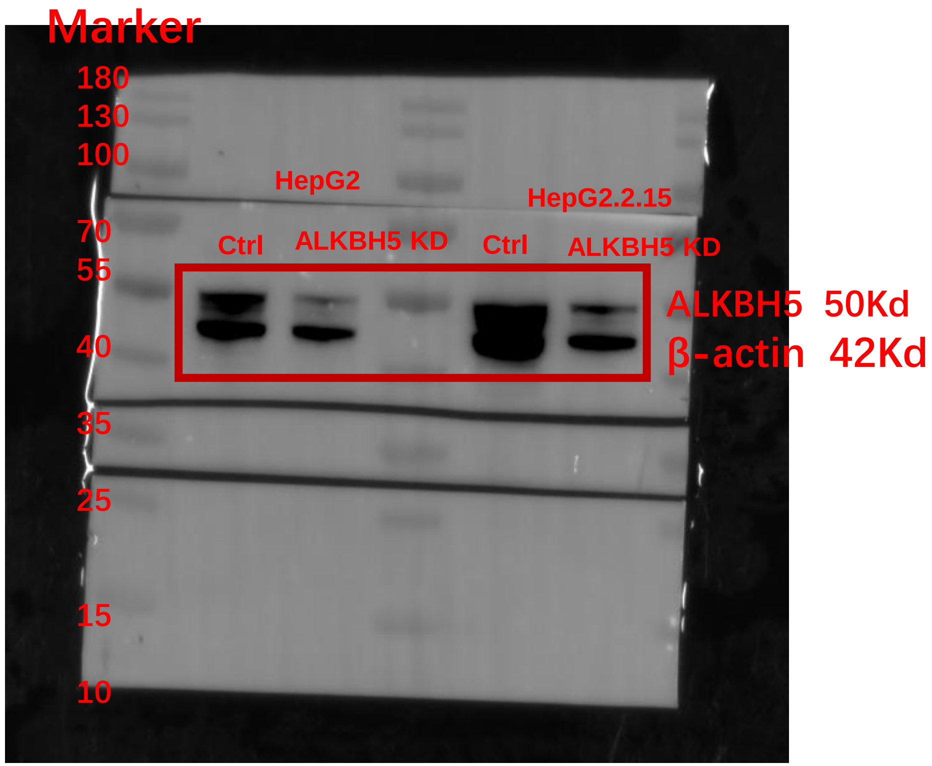
Marker
180
130
100
70
55
40
35
25
15
10
HepG2
HepG2.2.15
Ctrl
ALKBH5 KD
Ctrl
ALKBH5 KD
ALKBH5 50Kd
β-actin 42Kd

Figure 6F

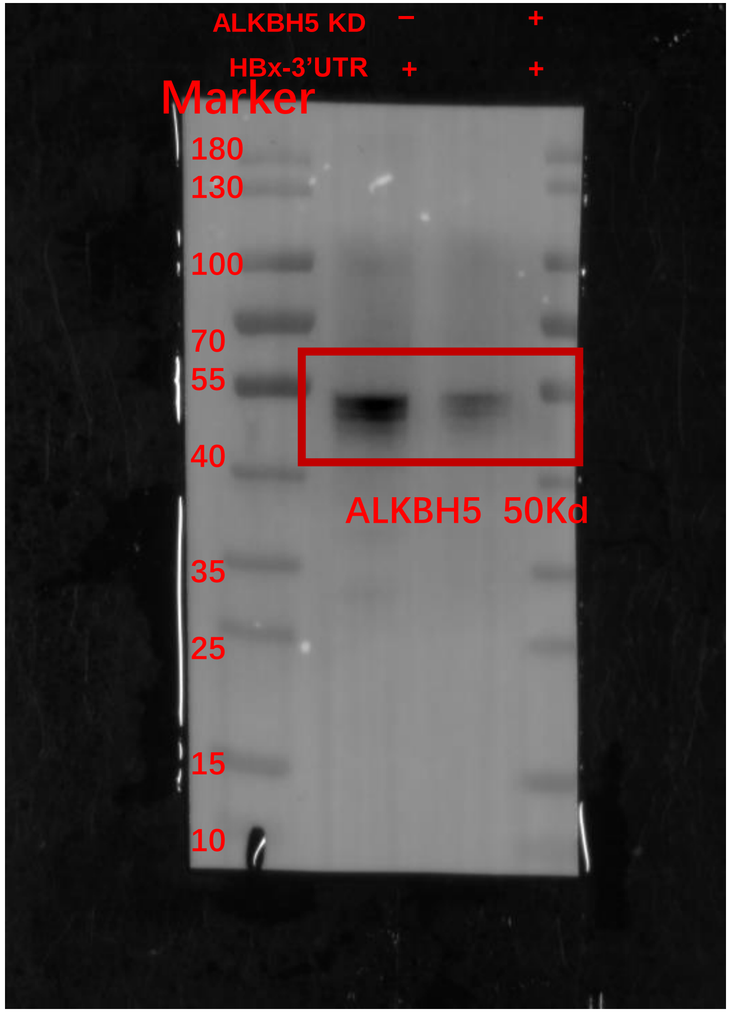

ALKBH5 KD – +
HBx-3'UTR + +
Marker
180
130
100
70
55
40
ALKBH5 50Kd
35
25
15
10

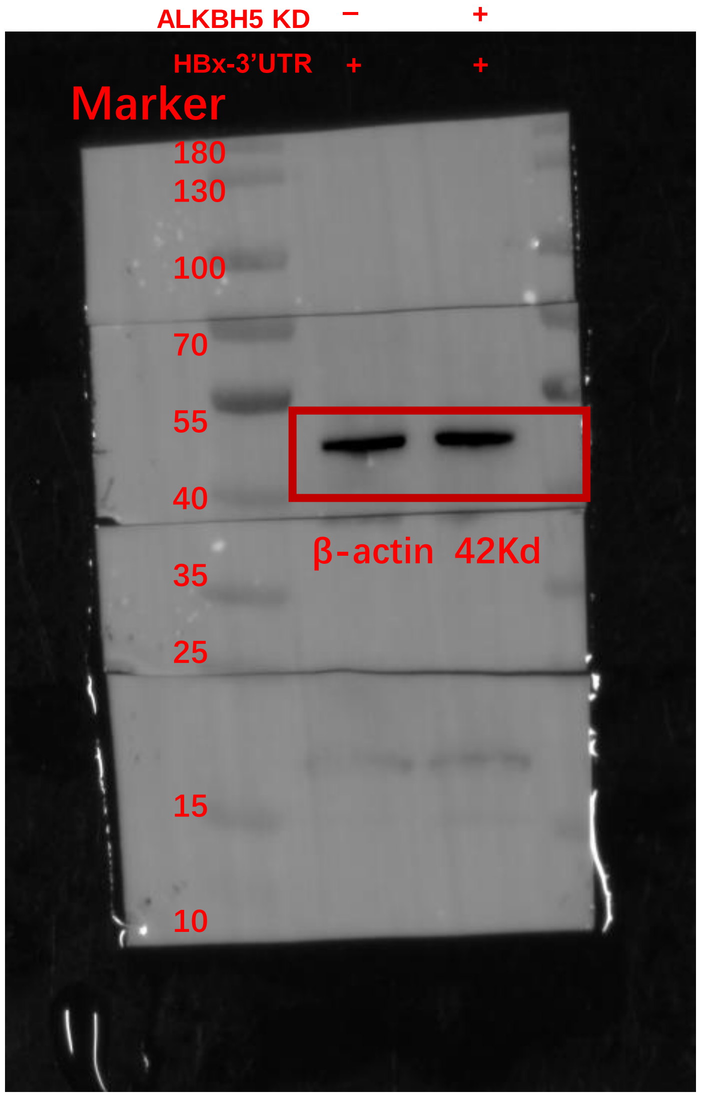

ALKBH5 KD – +
HBx-3'UTR + +
Marker
180
130
100
70
55
40
β-actin 42Kd
35
25
15
10

Figure 6F

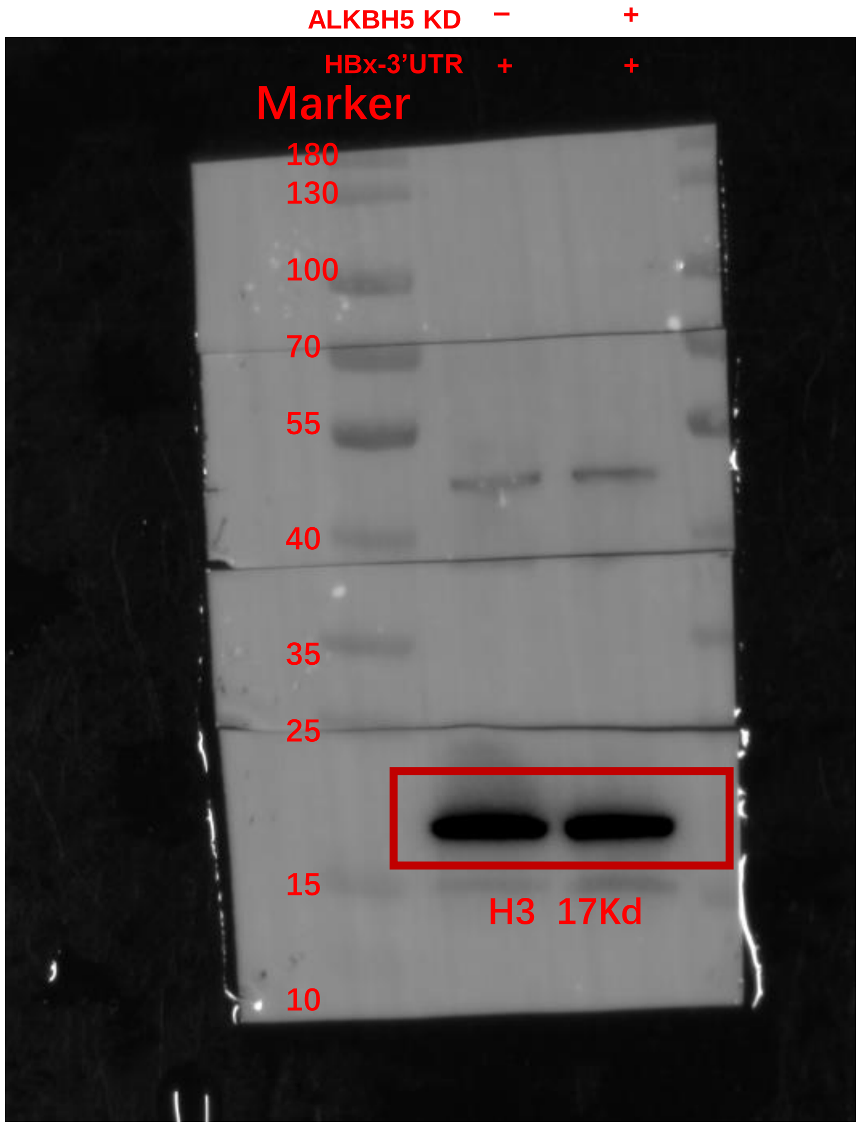
ALKBH5 KD − +
HBx-3'UTR + +
Marker
180
130
100
70
55
40
35
25
15
10
H3 17Kd

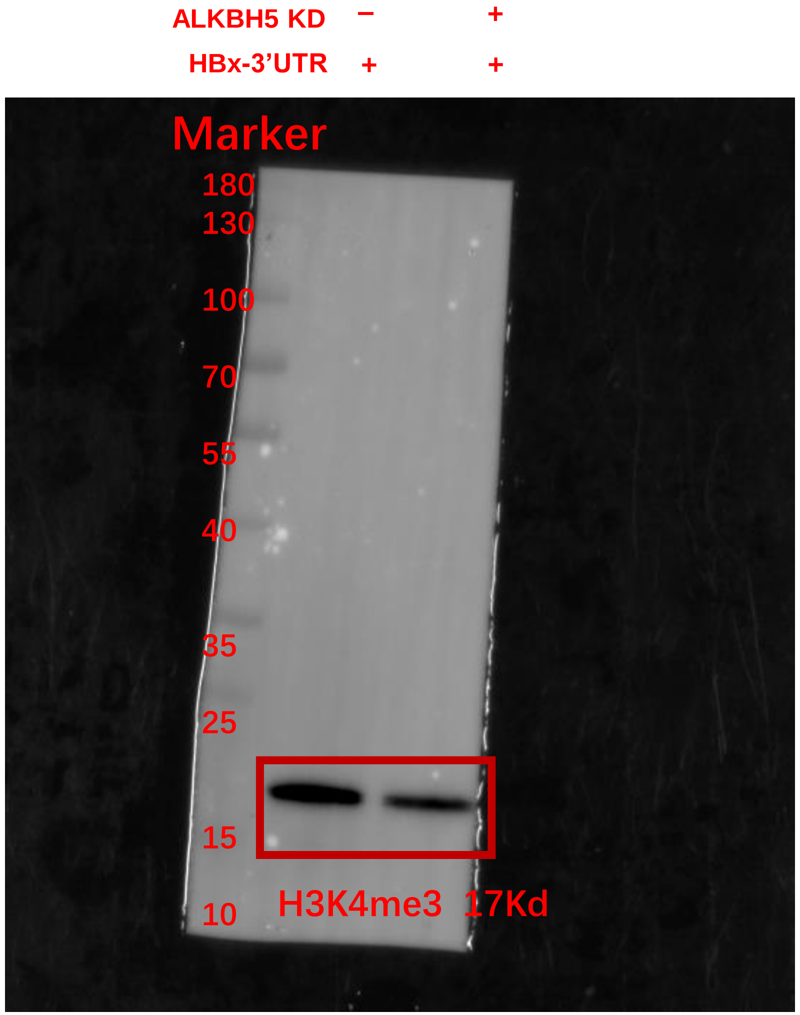
ALKBH5 KD − +
HBx-3'UTR + +
Marker
180
130
100
70
55
40
35
25
15
10
H3K4me3 17Kd

Figure 6F

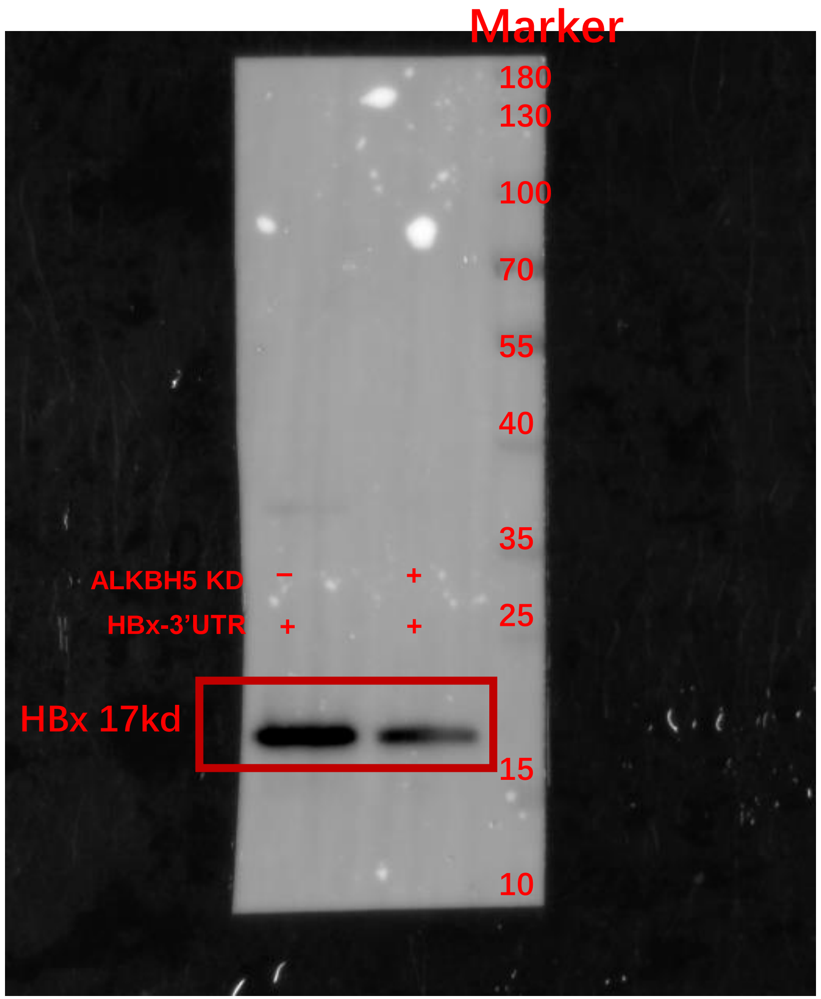
Marker
180
130
100
70
55
40
35
25
15
10
ALKBH5 KD − +
HBx-3'UTR + +
HBx 17kd

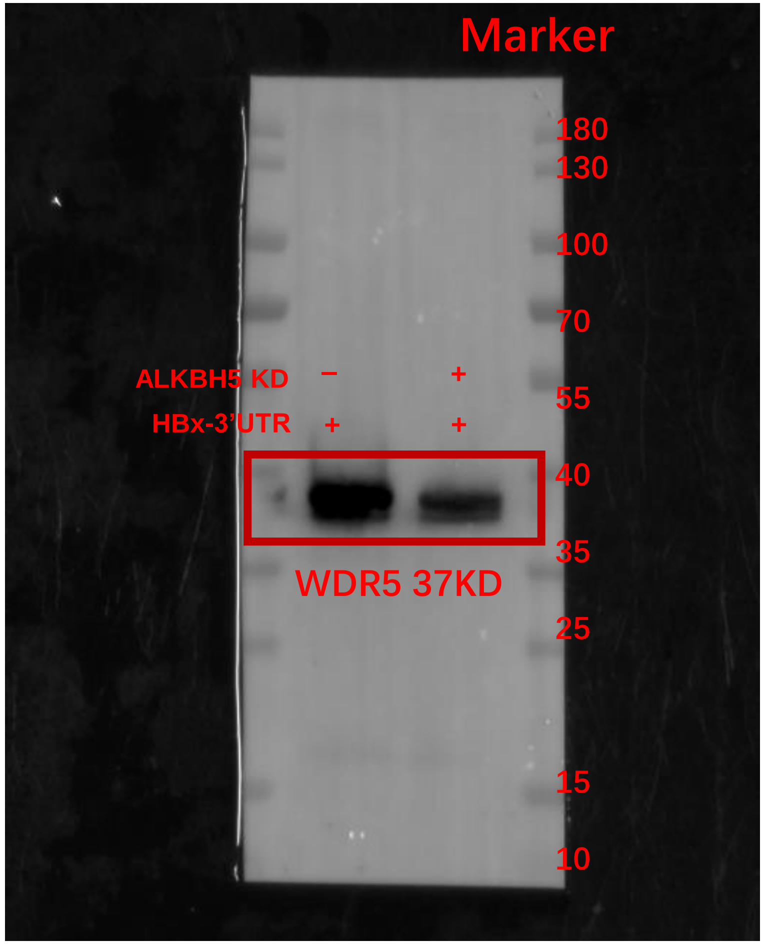
Marker
180
130
100
70
55
40
35
25
15
10
ALKBH5 KD − +
HBx-3'UTR + +
WDR5 37KD
